# One-step removal of alkynes and propadiene from cracking gases using a multi-functional molecular separator

**DOI:** 10.1038/s41467-022-30408-2

**Published:** 2022-05-26

**Authors:** Qingju Wang, Jianbo Hu, Lifeng Yang, Zhaoqiang Zhang, Tian Ke, Xili Cui, Huabin Xing

**Affiliations:** 1 Key Laboratory of Biomass Chemical Engineering of Ministry of Education, College of Chemical and Biological Engineering, Zhejiang University, Hangzhou, 310027 China; 2 ZJU-Hangzhou Global Scientific and Technological Innovation Center, Zhejiang University, Hangzhou, 311215 China

**Keywords:** Chemical engineering, Metal-organic frameworks, Crude oil

## Abstract

Refineries generally employ multiple energy-intensive distillation/adsorption columns to separate and purify complicated chemical mixtures. Materials such as multi-functional molecular separators integrating various modules capable of separating molecules according to their shape and chemical properties simultaneously may represent an alternative. Herein, we address this challenge in the context of one-step removal of alkynes and propadiene from cracking gases (up to 10 components) using a multi-functional and responsive material ZU-33 through a guest/temperature dual-response regulation strategy. The responsive and guest-adaptive properties of ZU-33 provide the optimized binding energy for alkynes and propadiene, and avoid the competitive adsorption of olefins and paraffins, which is verified by breakthrough tests, single-crystal X-ray diffraction experiments, and simulation studies. The responsive properties to different stimuli endow materials with multiple regulation methods and broaden the boundaries of the applicability of porous materials to challenging separations.

## Abstract (web summary)

Separating mixtures of hydrocarbons of low molecular weight is desirable but challenging. Here, the authors report a porous material with responsive and self-adaptive properties that enables one-step removal of alkynes and propadiene from cracking gases.

## Introduction

The discovery of synthetic zeolites^1–3^, metal-organic frameworks (MOFs)^4–6^, and other custom-designed porous materials^7–11^ opens the door to the successful adsorptive separation of chemical mixtures, depending on molecular structure rather than boiling points^12–17^. Recent advances in the ability to tailor pore chemistry in porous materials have broadened their separation range, according to the difference in molecular size/shape^18–20^, physicochemical properties^21–25^, or diffusion^26–28^ between the components. However, the industrial separation processes typically contain complicated multi-component mixtures with close even overlapped molecule size and properties, presenting a great challenge to the precise regulation and management of the pore chemistry of porous materials. For example, olefins (ethylene, propylene, 1,3-butadiene) are produced by separation and purification from the products of steam pyrolysis or cracking that include hydrogen (H_2_), methane (CH_4_), ethane (C_2_H_6_), ethylene (C_2_H_4_), acetylene (C_2_H_2_), propylene (C_3_H_6_), propyne (C_3_H_4_), propadiene (C_3_H_4_ (PD)), 1,3-butadiene (C_4_H_6_), *n*-butene (*n*-C_4_H_8_), *iso*-butene (*i*-C_4_H_8_) and etc.^29,30^. To obtain polymer-grade olefins, impurities of alkynes (acetylene and propyne) and propadiene must be removed from the cracking gases. Currently, complicated processes of multistep distillation and catalytic-hydrogenation units (Supplementary Fig. 1) are employed and cause large energy footprint^31,32^. For energy-efficient adsorption methods, most of the previous adsorbents were limited to the separation of the simulated two-component (C_2_H_2_/C_2_H_4_^33–35^, C_3_H_4_/C_3_H_6_^36–38^) or three-component mixtures (C_3_H_4_/C_3_H_4_ (PD)/C_3_H_6_)^39,40^. All these adsorbents failed to address the challenging one-step removal of alkynes and propadiene from complicated hydrocarbons mixtures (more than ten components). One-step purification of the target component from a complex mixtures is the most desired way for reducing the energy consumption and simplifying the separation process. Recently, the one-step production of polymer-grade C_2_H_4_ from a quaternary (CO_2_/C_2_H_2_/C_2_H_6_/C_2_H_4_) gas mixture has been investigated, yet using a series of adsorbents that each with selectivity for one of the impurities^41^. Therefore, it is urgent to develop new separation strategies and novel functional materials to deal with complex chemical mixtures.

**Fig. 1:**
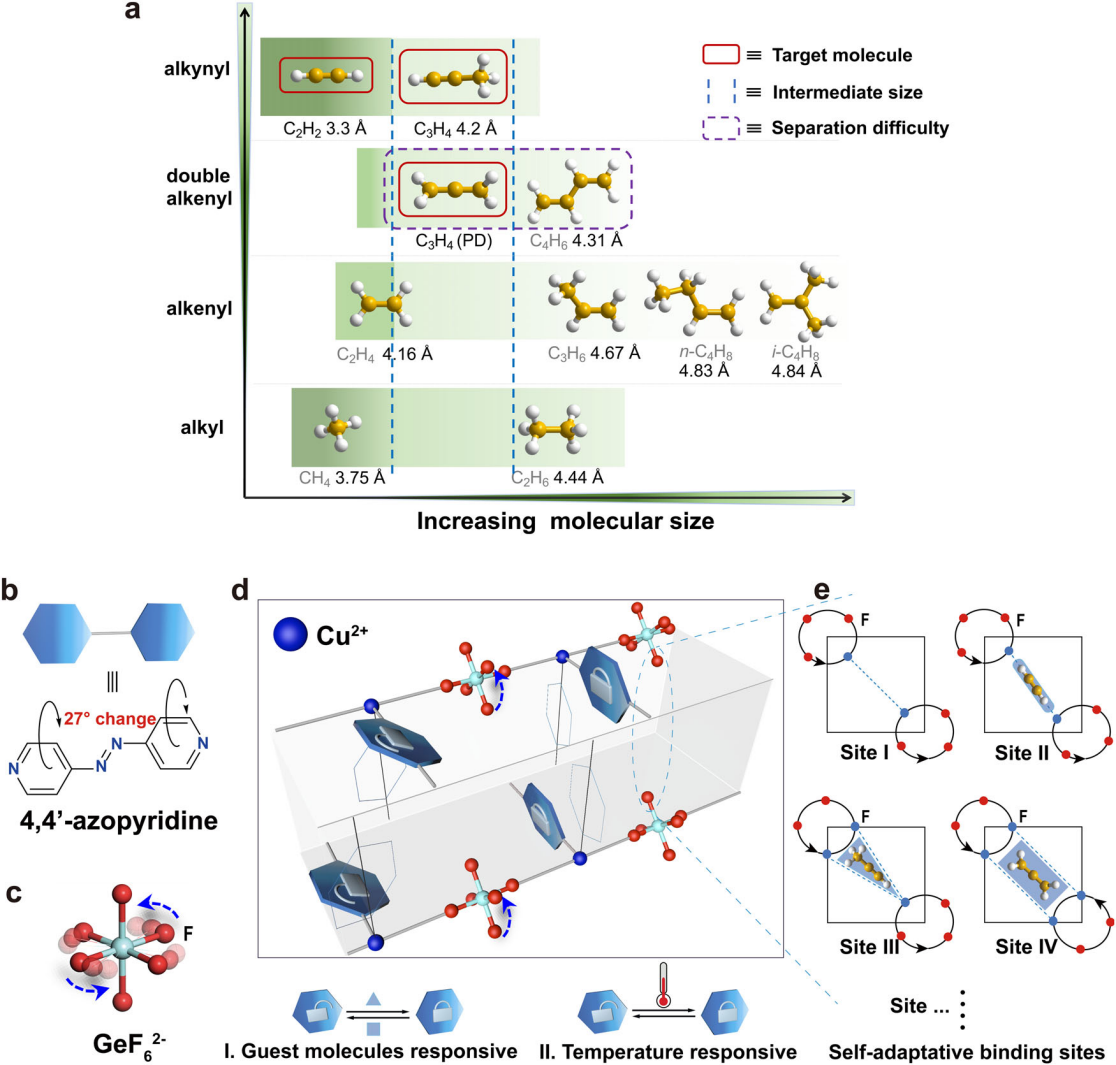
Properties of the hydrocarbons in cracking gases and separation mechanism of multi-functional ZU-33. **a** The kinetic diameters and functional groups of the hydrocarbons in cracking gases. The rotational units of pyridine rings (**b**) and F anions (**c**). **d** The guest/temperature dual-responsive mechanism of multi-functional ZU-33. **e** Schematic illustration of the adaptive binding sites in ZU-33. Color code: F, red; Ge, cyan; Cu, light blue; C (in hydrocarbons), orange; H, withe; N, blue.

A promising strategy would be to design a kind of multi-functional molecular separator that integrates multiple modules capable of separating molecules according to their size/shape and physical properties at the same time. The major barriers in the removal of alkynes and propadiene from cracking gases are: (i) the intermediate molecular sizes of target propyne and propadiene make the molecular sieving be never adaptable; (ii) the similar unsaturated bond structure, for example, propadiene and 1,3-butadiene show similar π–π conjugation effect which raises a strict challenge to achieve the separation via binding affinity difference (Fig. 1a); (iii) the deep and simultaneous removal of all three impurities (acetylene, propyne, and propadiene) with different structure and properties using one porous material.

In this work, we address the challenging task of alkynes and propadiene removal from cracking gases by designing porous materials that integrate synergistic suite of pore structures in one porous material to capture acetylene, propyne, and propadiene in one step. Inspired by the fact that rotational and responsive characters of building blocks offered the possibility of MOFs with multiple pore structures^42–47^, a multi-functional adsorbent (ZU-33, also termed as GeFSIX-14-Cu-i, GeFSIX = hexafluorogermanate, 14 = 4,4′- azopyridine) is constructed by the coordination of Cu ion with 4,4′-azobipyridne and then pillared by GeF_6_^2−^ anions. As noted previously, inorganic anions exhibite preferential binding of C_2_H_2_ molecules^24^. The rotation freedom of GeF_6_^2−^ anions and 4,4′-azobipyridne provide ZU-33 with the ability of responding accordingly to optimize the binding affinity towards alkynes and propadiene. Interestingly, the responsive properties of ZU-33 can be finely regulated by external temperatures and guest molecules. Modeling simulations indicate that alkenes and alkanes showed limited binding affinity with the pyridine-based pore window and cannot induce enough structural changes of the aperture, which causes the exclusion of alkenes and alkanes under relevant pressures. Meanwhile, the control of the rotation of 4,4′-azobipyridne by external stimulus of temperatures enables the efficient discrimination and separation between propadiene and 1,3-butadiene. Dynamic breakthrough experiments based on the multi-component cracking gas mixtures demonstrate the recognition and separation ability of ZU-33 for the deep removal of trace alkynes and propadiene.

## Results

The three-dimensional networks of ZU-33 (ZU = Zhejiang University) are constructed by the two-dimensional nets of 4,4′-azobipyridne and copper node which are further pillared with anions (GeF_6_^2−^) in the third dimension (Fig. 1 and Supplementary Fig. S3). Meanwhile, the independent nets are staggered with respect to one another to form doubly interpenetrated structures. As shown in Fig. 1d, the building ligands of GeF_6_^2−^ anion and 4,4′-azobipyridne are coordinated by the endpoint elements of N and F, endowing ZU-33 with rotational flexibility and responsive properties, which play a key role in recognizing target alkynes and propadiene. Interestingly, GeF_6_^2−^ anions and 4,4′-azobipyridne linkers are found to respond accordingly to the adsorption of guest molecules, offering a synergistic suite of pore structures (Fig. 1e) to optimize the guest-host interactions. The channel window of ZU-33 is relevant to the rotation angel of 4,4′-azobipyridne, ranging from 3.08 to 5.04 Å (Supplementary Fig. 4). Besides, the rotation properties of GeF_6_^2−^ anions and 4,4′-azobipyridne linkers can be fine-tuned by changing the temperature. The responsive characteristic of ZU-33 with diversified pore size and chemistry make it a potential candidate to handle complicated components.

**Fig. 2:**
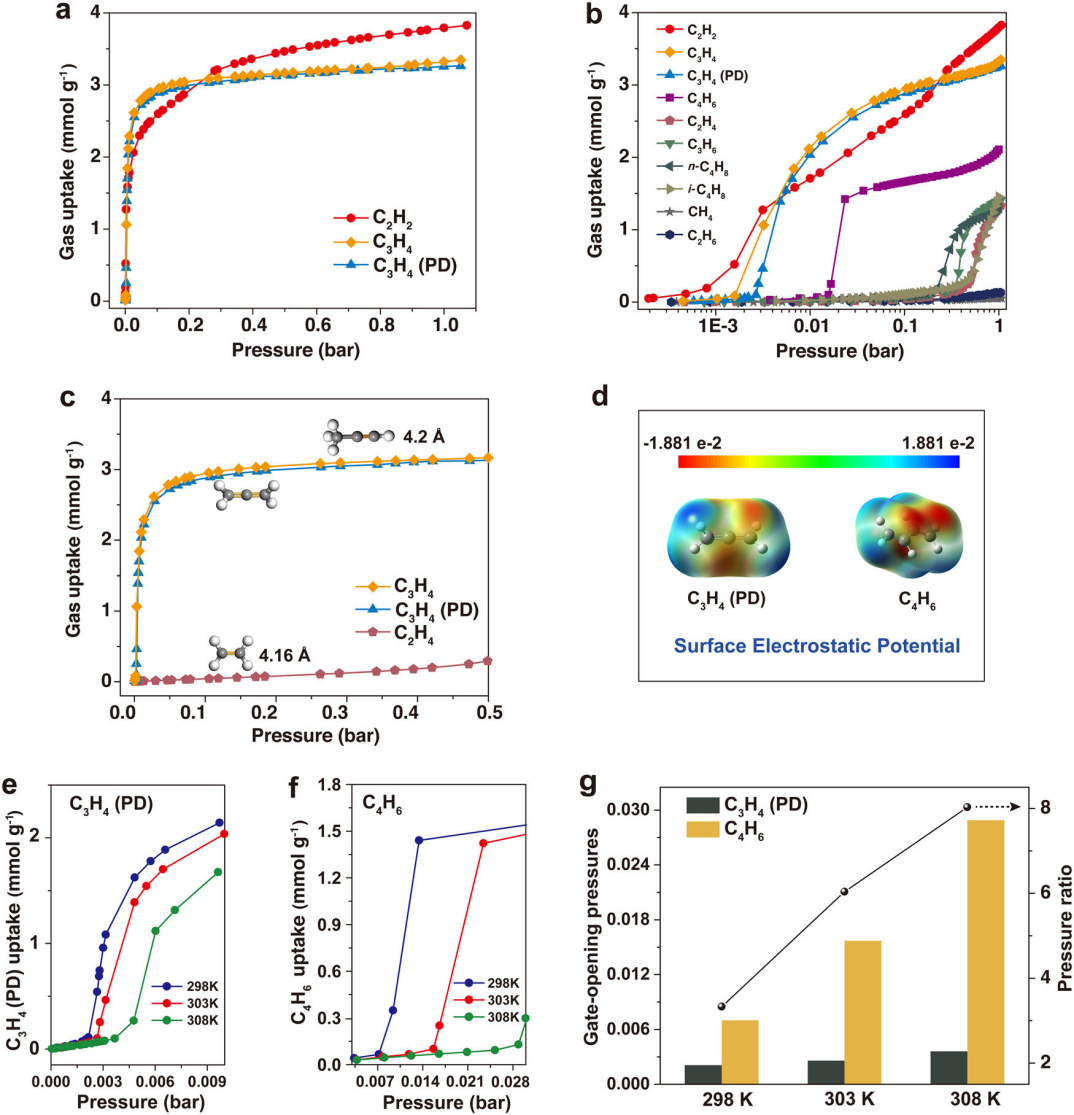
Single-component gas adsorption properties of ZU-33. **a** Adsorption isotherms for alkynes and propadiene (C_2_H_2_, C_3_H_4_, and C_3_H_4_ (PD)) at 303 K under 0–1.0 bar. **b** Adsorption isotherms for cracking gases (CH_4_, C_2_H_2_, C_2_H_4_, C_2_H_6_, C_3_H_4_, C_3_H_4_ (PD), C_3_H_6_, C_4_H_6_, *n*-C_4_H_8_, and *i*-C_4_H_8_) at 303 K under 0–1.0 bar. **c** Adsorption isotherms for C_2_H_4_ and C_3_H_4_, C_3_H_4_ (PD) with the intermediate molecular size at 303 K under 0–0.5 bar. **d** Surface electrostatic potential of C_3_H_4_ (PD) and C_4_H_6_ (red regions are negative ESP, and blue regions are positive ESP). Adsorption isotherms for C_3_H_4_ (PD) (**e**) and C_4_H_6_ (**f**) under low pressures at 298, 303, and 308 K. **g** Gate-opening pressures for C_3_H_4_ (PD) and C_4_H_6_ at different temperatures and the corresponding pressure ratios.

Single-component gas adsorption measurements were conducted at 298, 303, and 313 K (Supplementary Figs. 6–13) to explore the separation potential of ZU-33 towards the multi-component cracking gas mixtures. As depicted in Fig. 2a, the adsorption curves of the alkynes (acetylene, propyne) and propadiene on ZU-33 were steep at the low-pressure region. At 0.01 bar and 303 K, the adsorption capacities of ZU-33 for acetylene, propyne, and propadiene reached 1.7 mmol g^−1^ (C_2_H_2_), 2.2 mmol g^−1^ (C_3_H_4_), and 2.0 mmol g^−1^ (C_3_H_4_ (PD)), respectively, indicating the adsorption potential of ZU-33 for all alkynes and propadiene. In comparison, the obvious gate-opening behaviors were observed for the alkenes (ethylene, propylene, *n*-butene, and *iso*-butene) (Fig. 2b). For the adsorption of alkenes, the minimal threshold pressure was up to 0.2 bar, and the pressure for ethylene adsorption further increased to 0.5 bar at 303 K. Although the kinetic diameter of C_2_H_4_ (4.16 Å) was smaller than that of C_3_H_4_ (4.2 Å) and C_3_H_4_ (PD), ZU-33 could prevent C_2_H_4_ diffuse into the pore, realizing the inverse size sieving at 303 K and below 0.5 bar (Fig. 2c). The exclusion effect could be attributed to the weak binding affinity between the alkenes and ZU-33. As for the alkanes, ZU-33 showed molecular size sieving for CH_4_ and C_2_H_6_ at 303 K. The adsorption results demonstrated that, at a certain temperature and pressure range, ZU-33 showed high binding affinity for all target alkynes and propadiene with intermediate molecular sizes, meanwhile the desired exclusion effect to alkenes and alkanes in relevant pressures. Since the similar *π*–*π* conjugation effect and close molecular size between propadiene and 1,3-butadiene (Figs. 1a and 2d), it was difficult to realize obvious exclusion of 1,3-butadiene from propadiene. Upon controlling the temperature (298–308 K), the adsorption behavior of 1,3-butadiene and propadiene could be finely tuned. As shown in Fig. 2e, f the gate-opening pressures for 1,3-butadiene showed a significant shift, from 0.007 bar at 298 K to 0.028 bar at 308 K, with an increase of 300, while that of propadiene just increased by 75%, from 0.002 to 0.0035 bar. Correspondingly, the gate-opening pressure ratio of ${{{{{{\rm{P}}}}}}}_{{{{{{{\rm{C}}}}}}}_{4}}{{{{{{\rm{H}}}}}}}_{6}/{{{{{{\rm{P}}}}}}}_{{{{{{{\rm{C}}}}}}}_{3}}{{{{{{\rm{H}}}}}}}_{4}$_(PD)_ increased from 3.5 (298 K) to 8 (308 K) (Fig. 2g). Adsorption results indicated that the adsorption of 1,3-butadiene was more sensitive to the temperature than that of propadiene, providing the chance to achieve a distinct separation between 1,3-butadiene and propadiene via the fine-tuning of temperatures. Therefore, ZU-33 showed potential in the selective capture of alkynes and propadiene with high binding affinity, while effectively excluded other hydrocarbons at relevant pressures, rendering it an ideal candidate for the one-step removal of alkynes and propadiene from the cracking gases.

**Fig. 3:**
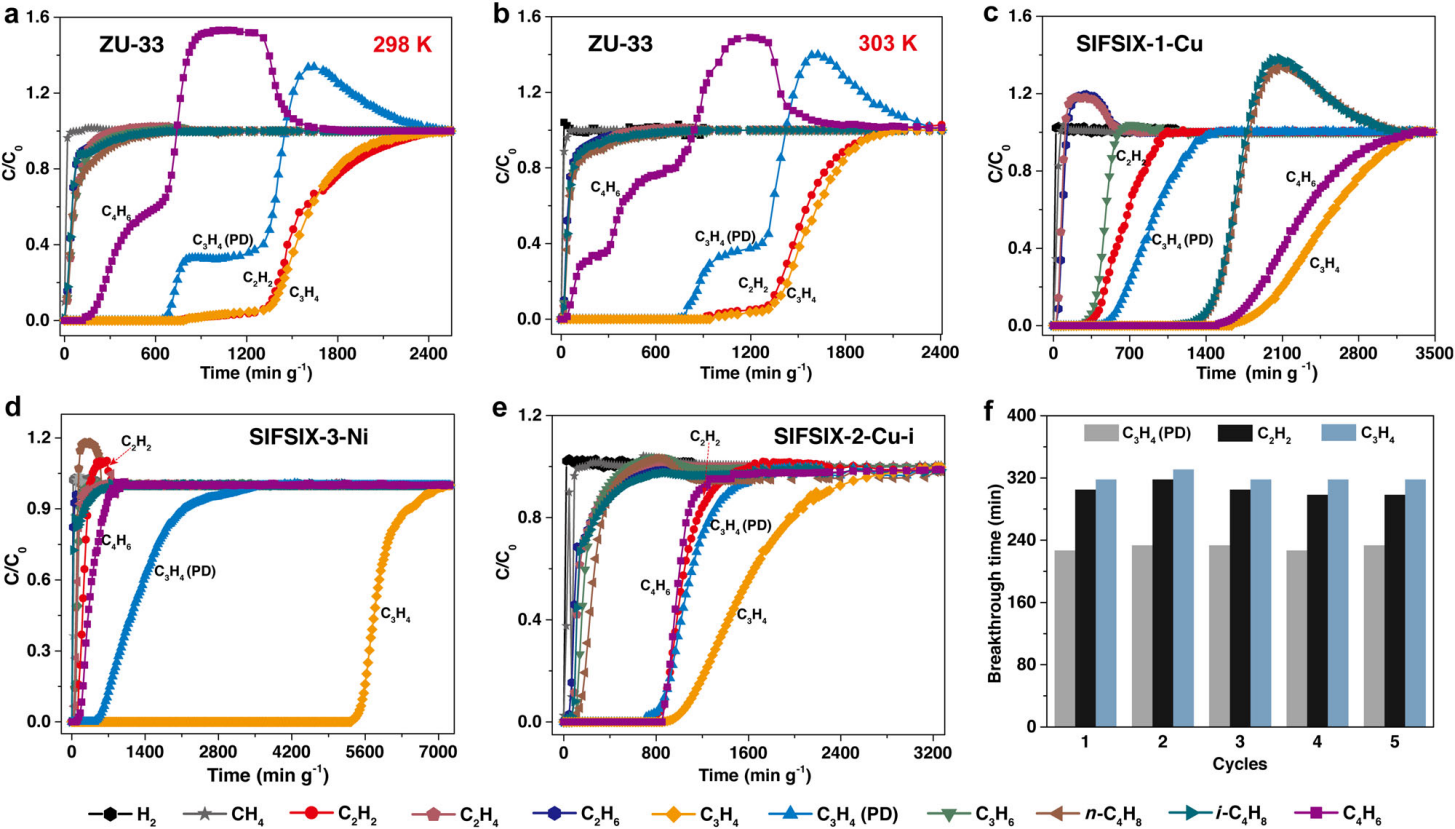
Mixture separation performance. Experimental column breakthrough curves for the mixture of H_2_/CH_4_/C_2_H_2_/C_2_H_4_/C_2_H_6_/C_3_H_4_/C_3_H_4_ (PD)/C_3_H_6_/C_4_H_6_/*n*-C_4_H_8_/*i*-C_4_H_8_ on ZU-33 at 298 K (**a**), 303 K (**b**), SIFSIX-1-Cu (**c**), SIFSIX-3-Ni (**d**) and SIFSIX-2-Cu-i (**e**) at 298 K. **f** Cycling breakthrough tests on ZU-33 at 303 K. *C*/*C*_0_, outlet concentration/feed concentration.

To confirm the separation ability of ZU-33, the dynamic breakthrough experiments were conducted at various temperatures. To mimic the cracking gases, a multi-component mixture including 15.57% hydrogen, 30% methane, 1.02% acetylene, 34.3% ethylene, 6.99% ethane, 0.3% propyne, 0.28% propadiene, 8.93% propylene, 1.48% 1,3-butadiene, 0.42% *n*-butene and 0.71% *iso*-butene was obtained^48^. The breakthrough experiments were conducted with the mixed gas passing through the column packed on the activated ZU-33. As illustrated in Fig. 3a, at 298 K, hydrogen, alkanes, and alkenes broke through immediately, whereas the alkynes and propadiene were retained in the packed column over a *t*_m_ of 215 min (*t*_m_ was defined as the maximal breakthrough time with the outlet concentration of each impurity was less than 1 ppm). However, 1,3-butadiene was also adsorbed, and the retained time was up to 50 min. Considering that the adsorption of 1,3-butadiene on ZU-33 was sensitive to the temperature as revealed by the adsorption isotherms results, we attempted to improve the separation performance by adjusting the temperature. When tuning the temperature to 303 K (Fig. 3b), an optimized separation performance was observed with hydrogen, alkanes, alkenes, and 1,3-butadiene eluted through the packed column immediately, only the alkynes and propadiene were retained in the packed column with a *t*_m_ of 233 min, indicating the successful one-step removal of alkynes and propadiene using ZU-33. The working capacities of acetylene, propyne, and propadiene were about 1.53 mmol g^−1^, 0.39 mmol g^−1^, 0.23 mmol g^−1^, respectively. In contrast, all the other adsorbents of SIFSIX-1-Cu (Fig. 3c), SIFSIX-3-Ni (Fig. 3d), SIFSIX-2-Cu-i (Fig. 3e), and TIFSIX-2-Cu-i (Supplementary Fig. 17) exhibited comparable adsorption of alkenes or 1,3-butadiene that should be recovered, and showed poor separation performance for the simultaneous removal of acetylene, propyne, and propadiene from cracking gases. In addition, the cycling ability and regeneration condition of ZU-33 were further investigated by breakthrough tests (Fig. 3f and Supplementary Fig. 18), revealing its excellent recyclability. And carbon dioxide (400–1000 ppm), moisture (400–1000 ppm), oxygen, nitrogen do not affect the alkynes and propadiene capture ability of ZU-33 (Supplementary Figs. 14, 15, 19, and 20). Therefore, combined with the fine regulation of temperatures, ZU-33 achieved the challenging one-step removal of alkynes and propadiene form cracking gases under mild conditions (1 bar, 303 K).

**Fig. 4:**
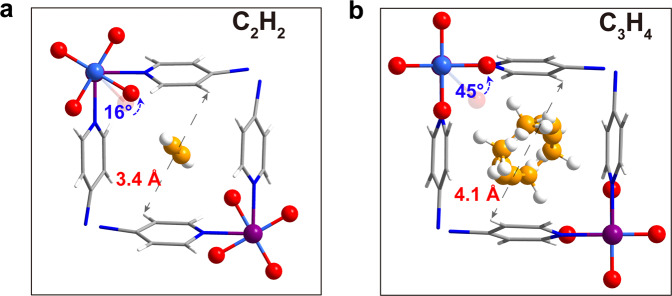
X-ray crystal of guest-loaded ZU-33. Single-crystal structure of ZU-33·C_2_H_2_ (**a**), ZU-33·C_3_H_4_ (**b**). Color code: F, red; Ge, light blue; Cu, modena; C (in framework), gray; C (in hydrocarbons), orange; H, withe; N, blue.

To investigate the guest-responsive properties and pore structures of ZU-33, single-crystal X-ray diffraction (SCXRD) measurements were performed on alkynes-loaded ZU-33 crystals. As depicted in Fig. 4a, when loaded with C_2_H_2_ molecules, the pyridine rings of azpy showed subtle rotation (about 3°), and the corresponding pore size was 3.4 Å. The exposed F of GeF_6_^2−^ anions rotated from 45 to 29° to match the C_2_H_2_ molecules well. While in the C_3_H_4_-loaded structure (Fig. 4b), the pyridine rings exhibited a visible rotation from ca. 27 to 16°, this noticeable degree change induced a significant structural transformation from narrow (3.0 Å) to expanded pores (4.1 Å), allowing the passing of the lager-size guest, and the uncoordinated F atoms showed an obvious rotation (from 45 to 0°) to adapt the shape/size of C_3_H_4_ molecules for the most energy favorable binding sites. The different conformations when loaded with different alkynes confirmed the guest-adaptive properties of ZU-33 and further elucidated the strong binding affinity for all alkynes and propadiene with ZU-33.

**Fig. 5:**
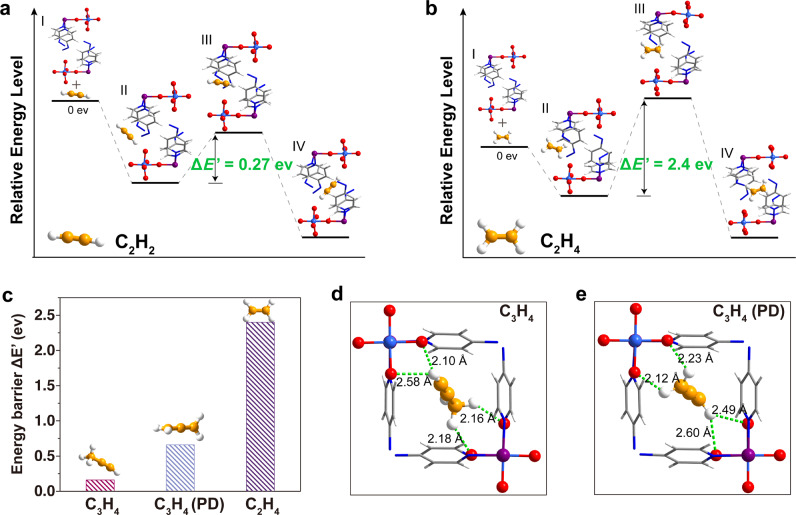
The energy landscape of the host–guest conformations, DFT-D structures of guest-loaded ZU-33. Illustration of the interaction energy pathway and the corresponding energy levels calculated by DFT for C_2_H_2_ (**a**), C_2_H_4_ (**b**). **c** Comparison of energy barriers of C_2_H_4_, C_3_H_4_, and C_3_H_4_ (PD). DFT-D calculated adsorption binding sites for C_3_H_4_ (**d**), C_3_H_4_ (PD) (**e**). Color code: F, red; Ge, light blue; Cu, modena; C (in framework), gray; C (in hydrocarbons), orange; H, withe; N, blue.

Furthermore, molecular simulation was employed to reveal the separating mechanism of the molecular separator, and the detailed simulation methods were introduced in the Supplementary Information. The whole adsorption process was rationally classified into several states as shown in Fig. 5a, b. In the initial state, guest molecules and host framework were independent of each other, and the system energy was set as the reference (state I, the optimized guest-free host and optimized guest). Then, guest molecules gradually diffused onto the host surface (state II), further moved into the channel, and finally were firmly trapped in the cavity, reaching the minimum of conformational energy landscape (state IV, the optimized host–guest structures). Flexible deformations occurred in the host framework during the process of guest molecules migrating from the surface to the most stable state, in which the highest energy level of the system was the transition state (state III). The energy barrier (Δ*E*′) was defined as the energy difference between the state II and state III, a quantitative value that assessed the permeation ease of the probe molecule into the channel^49,50^. Guest molecules, such as C_2_H_2_ and C_2_H_4_ with prominent difference in molecular size, exhibited significantly different energy barriers, and the values were 0.27 eV (C_2_H_2_, 3.3 Å) and 2.4 eV, (C_2_H_4_, 4.16 Å), respectively (Fig. 5a, b). These results indicated the greater resistance for C_2_H_4_ molecules to diffuse into the pore of ZU-33 compared with C_2_H_2_, which was consistent with the higher gate-opening pressure of C_2_H_4_ shown in the adsorption isotherms. The C_2_H_4_ molecule with larger molecular size could make greater deformation of the host-framework, and more input energy would be compensated to allow the adsorption of C_2_H_4_ molecules, thereby resulting in the higher energy barrier. As for the guest molecules with similar size, such as C_3_H_4_ (4.2 Å), C_3_H_4_ (PD), and C_2_H_4_ (4.16 Å), the energy barriers were in the order Δ*E*′(C_3_H_4_) < Δ*E*′(C_3_H_4_ (PD)) < Δ*E*′(C_2_H_4_) (Fig. 5c and Supplementary Fig. 22), which was also well agreement with the order of gate-opening pressures shown in the adsorption isotherms. Guest molecules with larger dipole moment and/or polarizability could interact more strongly with the framework and induce the structural deformation more easily^42,43,50^, thus corresponded to the lower energy barriers. These results also revealed the reason for the inverse phenomenon that C_3_H_4_ and C_3_H_4_ (PD) with large molecular size and larger dipole moment and/or polarizability than C_2_H_4_, but the almost negligible gate-opening pressures. The selective admission of hydrocarbons by the guest-responsive mechanism was revealed by the simulation model, and the molecular size and properties were the two main factors to synergistically affect the energy barriers, further determining the threshold pressures of different guests.

Moreover, the alkynes and propadiene adsorption behaviors (state IV) on ZU-33 were further determined by the first-principles dispersion-corrected density functional theory (DFT-D) method. The obtained host–guest structures exhibited the same trend as the above-mentioned SCXRD experiment results. As shown in Fig. 5d, e, the structure deformed to fit the guests after accommodating C_3_H_4_ and C_3_H_4_ (PD) molecules. The pyridine rings of azpy and the exposed F atoms rotated about 12 and 45° when loaded with C_3_H_4_ molecules, which were consistent with the SCXRD results. Each adsorbed C_3_H_4_ was bound with two GeF_6_^2−^ sites through cooperative C≡H···F and C-H···F binding with a distance ranged from 2.10 to 2.58 Å (Fig. 5d), and the DFT-D-calculated static adsorption energy (Δ*E*) was 62.49 kJ mol^−1^. Similarly, each C_3_H_4_ (PD) molecule was bound with two GeF_6_^2−^ sites through multiple C=H···F (2.12–2.60 Å), and the corresponding static adsorption energy (Δ*E*) was 53.76 kJ mol^−1^ (Fig. 5e). The C_2_H_2_ molecule was trapped through two uncoordinated F atoms from GeF_6_^2−^ anions of different nets, the corresponding H-bonding distance between C≡H···F was 1.89 Å, the adsorption energy (Δ*E*) was 52.08 kJ mol^−1^(Supplementary Fig. 23). The self-adaptive behavior of ZU-33 enabled the maximum strong interactions with all alkynes and propadiene, which enhanced the removal ability of ZU-33. Overall, the responsive characteristic enabled ZU-33 to exhibit various conformations when encountering different guest molecules, rendering it a multi-functional molecular separator integrated with diversified pore size and chemistry to address the challenging task.

## Discussion

In summary, a multi-functional molecular separator with integrated diversified pore size and chemistry was demonstrated to achieve the one-step removal of alkynes and propadiene from cracking gases. It is interesting to note that porous materials that can deal with such complicated mixtures with close and overlapped properties are rare. This work reveals the great advantage of responsive materials to deal with complicated hydrocarbon components, provides insight for the separation of the complex mixtures, and brings guidance for the design of the next-generation materials.

## Methods

### Preparation of ZU-33 (Cu(4,4′-azopyridine)_2_GeF_6_)

The synthesis of ZU-33 was referred to previous literature with some modifications^14^. The single crystals of ZU-33 were synthesized by slow diffusion of a methanol solution (4.0 mL) of (NH_4_)_2_GeF_6_ (0.011 g, 0.05 mmol) and Cu(BF_4_)_2_·*x*H_2_O (0.012 g, 0.05 mmol) into a DMSO solution (4.0 mL) of 4,4′-azopyridine (0.018 g, 0.1 mmol) after one week.

#### Powder synthetic method 1

A methanol solution (8.0 mL) of 4,4′-azopyridine (0.095 g, 0.5 mmol) was mixed with an aqueous solution (8.0 mL) of (NH_4_)_2_GeF_6_ (0.056 g, 0.25 mmol) and Cu(BF_4_)_2_·*x*H_2_O (0.059 g, 0.25 mmol). Then, the mixture was stirred at room temperature for 10 s. The obtained powder was filtered, washed with methanol, and exchanged with methanol for two days.

#### Powder synthetic method 2

A preheated methanol solution (8.0 mL) of 4,4′-azopyridine (0.095 g, 0.5 mmol) was dropped into a preheated methanol solution (8.0 mL) of (NH_4_)_2_GeF_6_ (0.056 g, 0.25 mmol) and Cu(BF_4_)_2_·*x*H_2_O (0.059 g, 0.25 mmol). Then, the mixture was heated at 65 °C for 24 h under stirring. The obtained powder was filtered, washed with methanol, and exchanged with methanol for two days.

### Gas adsorption measurements

ZU-33 was evacuated at 65 °C for 12 h until the pressure dropped below 7 µm Hg. The single-component adsorption isotherms of CH_4_, C_2_H_2_, C_2_H_4_, C_2_H_6_, C_3_H_4_, C_3_H_4_(PD), and C_3_H_6_ were collected at 298–308 K on activated ZU-33 using ASAP 2460 Analyzer (Micromeritics). The single-component adsorption isotherms of C_4_H_6_, *n*-C_4_H_8_, and *i*-C_4_H_8_ were collected at 298–308 K on activated ZU-33 using ASAP 2050 Analyzer (Micromeritics).

### Breakthrough experiments

The breakthrough experiments were carried out in a dynamic gas breakthrough equipment. All experiments were conducted using a stainless steel column (4.6 mm inner diameter × 50 mm). According to the different particle size and density of the sample powder, the weight packed in the column was: 0.332 g for ZU-33, 0.3 g for SIFSIX-1-Cu, 0.21 g for SIFSIX-2-Cu-i, 0.242 g for TIFSIX-2-Cu-i and 0.26 g for SIFSIX-3-Ni. The mixed gas of H_2_/CH_4_/C_2_H_2_/C_2_H_4_/C_2_H_6_/C_3_H_4_/C_3_H_4_ (PD)/C_3_H_6_/C_4_H_6_/*n*-C_4_H_8_/*i*-C_4_H_8_ = 15.57/30/1.02/34.3/6.99/0.3/0.28/8.93/1.48/0.42/0.71 (v/v/v/v/v/v/v/v/v/v/v) was introduced at a total flow rate of 2.0 mL min^−1^ at 298 and 303 K. Outlet gas from the column was monitored using gas chromatography (GC-490) with the thermal conductivity detector TCD. After breakthrough experiment, the adsorption bed was regenerated by N_2_ flow (10 mL min^−1^) for 1 h at 323 K or by vacuuming for about 30 min at 323 K.

### Single crystal X-ray diffraction

Single crystal X-ray diffraction data for ZU-33 were collected on a Bruker D8 VENTURE diffractometer equipped with a PHOTONII/CMOS detector (GaKα, λ = 1.314139 Å). Indexing was performed using APEX3. Data integration and reduction were completed using SaintPlus 6.01. Absorption correction was performed by the multi-scan method implemented in SADABS. The space group was determined using XPREP implemented in APEX3. The structure was solved with SHELXS-2018 (direct methods) and refined on F2(nonlinear least-squares method) with SHELXL-2018 contained in APEX3 program packages. All non-hydrogen atoms were refined anisotropically. Firstly, the ZU-33 single crystals were loaded into the sample tube for adsorption test, the inner wall of the sample tube was coated with oil (polybutenes), which slowly flowed down the tube wall. Then, C_2_H_2_ and C_3_H_4_ gas were backfilled, respectively. After ZU-33 single crystals were sealed by oil, they were taken out and tested on a Bruker D8 VENTURE diffractometer.

### Computational methods

#### Simulation details

All calculations were performed using the combination of first-principle density functional theory (DFT) and plane-wave ultrasoft pseudopotentil implemented in the Materials Studio, CASTEP code. A semi-empirical addition of dispersive forces to conventional DFT was included in the calculation to account for van der Waals interactions. Calculations were performed under the generalized gradient approximation (GGA) with Perdew-Burke-Ernzerhof (PBE) exchange correlation. A cutoff energy of 544 eV and a 2×2×4 k-point mesh with smearing 0.2 eV were found to be enough for the total energy to converge within 1 × 10^−6^ eV/atom. Notably, in all the simulation process, the structures were optimized with a full structural relaxation that allowed all atomic positions and unit cell parameters to vary.

#### The energy barrier

The periodic slab models with periodic boundary conditions were sued to represent ZU-33 surface. The vacuum region between slabs was 20 Å to eliminate spurious interactions between the guest molecules with the periodic image of the bottom layer of the surface. The surface model and the host structure would be first optimized using the experimentally-obtained single crystal structures as initial geometries with a full structural relaxation. The isolated guest molecules (C_2_H_2_, C_2_H_4_, C_3_H_4_, and C_3_H_4_ (PD)) were placed in a supercell (with the same cell dimensions as the MOF crystal) and also relaxed as references. Then, the guest molecules were introduced onto the host surface and different locations in the channel pore of the host structure, respectively, followed by a full structural relaxation. And the optimized configurations having the lowest energy were used for the subsequent analysis and calculation. The transition state search calculations were used to capture the transition statues associated with guest transport between the two know energy minimum configurations from the host surface to the channel pore.

The initial state I was defined as the optimized guest-free host and optimized guest, and the system energy was set as the reference. The state II was defined as the optimized host–guest structures where guests were introduced onto the host surface, the state III was defined as the transition state and the state IV was defined as the optimized host–guest structures where guests were introduced into the channel pore. The energy barrier was calculated using:

1 $$\varDelta E^{\prime}={E}({{{{{\rm{state}}}}}}\,{{{{{\rm{III}}}}}})-{E}({{{{{\rm{state}}}}}}\,{{{{{\rm{II}}}}}})$$

where *E* (state III) is the energy of the transition state, *E* (state II) is the energy of the optimized host–guest structures where guests were introduced onto the host surface.

#### The static binding energy

The static binding energy (at *T* = 0 K) was calculated using:

2 $$\bigtriangleup E={E}({{{{{\rm{MOF}}}}}})+{E}({{{{{\rm{gas}}}}}})-{E}({{{{{\rm{MOF}}}}}}+{{{{{\rm{gas}}}}}})$$

where *E* (MOF) is the energy of the optimized guest-free host, *E* (gas) is the energy of the optimized guest, and *E* (MOF + gas) is the total energy of the optimized host–guest structures.

## Supplementary information

Supplementary Information

## Data Availability

All data supporting the findings of this study are available within this article and its Supplementary Information. Crystallographic data for the structures in this article have been deposited at the Cambridge Crystallographic Data Center under deposition Nos. CCDC 2090065 (degassed ZU-33), 2090064 (C_2_H_2_-loaded ZU-33) and 2090063 (C_3_H_4_-loaded ZU-33). Copies of the data can be obtained free of charge from www.ccdc.cam.ac.uk/data_request/cif. Source data that support the findings of this study are available from the corresponding author upon request. Correspondence and requests for materials should be addressed to X.C. and H.X.
