# Supplementary material for: One-step removal of alkynes and propadiene from cracking gases using a multi-functional molecular separator
Source: Nat Commun. 2022 May 26;13:2955. doi: 10.1038/s41467-022-30408-2 (PMC9135742; doi:10.1038/s41467-022-30408-2)
Supplement: Supplementary file 1 — Supplementary Information [file 41467_2022_30408_MOESM1_ESM.pdf]

One-step removal of alkynes and propadiene from cracking gases using a multi-functional molecular separator

Qingju Wang,[1#] Jianbo Hu,[1,2#] Lifeng Yang,[1] Zhaoqiang Zhang,[1] Tian Ke,[1] Xili Cui,[1,2*] Huabin Xing[1,2*]

[1]Key Laboratory of Biomass Chemical Engineering of Ministry of Education, College of Chemical and Biological Engineering, Zhejiang University, Hangzhou 310027, China.

[2]ZJU-Hangzhou Global Scientific and Technological Innovation Center, Zhejiang University, Hangzhou 311215, China.

*To whom correspondence should be addressed. E-mails: cuixl@zju.edu.cn, xinghb@zju.edu.cn

Supplementary Methods

Materials and Reagents

All reagents and solvents were commercially purchased and used without further purification. Ammonium hexafluorogermanate ($(NH_4)_2GeF_6$, 99%, Aldrich), ammonium hexafluorosilicate ($(NH_4)_2SiF_6$, 98%, Aldrich), ammonium hexafluorotitanate ($(NH_4)_2TiF_6$, 99%, Aldrich), copper (II) tetrafluoroborate hydrate ($Cu(BF_4)_2 \cdot xH_2O$, 98%, Aldrich), nickel tetrafluoroborate hexahydrate ($Ni(BF_4)_2 \cdot 6H_2O$, 99%, Aldrich), (4-(2-pyridin-4-ylethynyl)pyridine ($C_{12}H_8N_2$, 98%, Chemsoon), pyrazine ($C_4H_4N_2$, 99%, Aldrich), 4,4′-azopyridine ($C_{10}H_8N_4$, 98%, Chemsoon), methanol (CH_3OH, anhydrous, 99%, Sinopharm), dimethyl sulfoxide (DMSO, 99%, Sinopharm), ethanol (C_2H_5OH, anhydrous, 99%, Sinopharm), ethylene glycol (99%, Sinopharm), Polybutenes (Aldrich).

N_2 (99.999%), He (99.999%), CH_4 (99.999%), C_2H_2 (99%), C_2H_4 (99.99%), C_2H_6 (99.999%), C_3H_4 (99.999%), C_3H_4 (PD) (99.999%), C_3H_6 (99.999%), C_4H_6 (99.999%), n-C_4H_8 (99.999%), i-C_4H_8 (99.999%) were purchased from JinGong Company. Mixed gases of $H_2/CH_4/C_2H_2/C_2H_4/C_2H_6/C_3H_4/C_3H_4(PD)/C_3H_6/C_4H_6/n\text{-}C_4H_8/i\text{-}C_4H_8$ = 15.57/30/1.02/34.3/6.99/0.3/0.28/8.93/1.48/0.42/0.71 (v/v/v/v/v/v/v/v/v/v/v) were purchased from Shanghai Wetry Standard Reference Gas Analytical Technology Co. LTD (China)

Synthesis of materials

Preparation of ZU-33 ($Cu(4,4'\text{-azopyridine})_2GeF_6$)

The synthesis of ZU-33 were referred to previous literature with some modifications[1]. The single crystals of ZU-33 were synthesized by slow diffusion of a methanol solution (4.0 mL) of $(NH_4)_2GeF_6$ (0.011 g, 0.05 mmol) and $Cu(BF_4)_2 \cdot xH_2O$ (0.012 g, 0.05 mmol) into a DMSO solution (4.0 mL) of 4,4'-azopyridine (0.018 g, 0.1 mmol) after one week.

Powder synthetic method 1: A methanol solution (8.0 mL) of 4,4′-azopyridine (0.095 g, 0.5 mmol) was mixed with an aqueous solution (8.0 mL) of $(NH_4)_2GeF_6$ (0.056 g, 0.25 mmol) and $Cu(BF_4)_2 \cdot xH_2O$ (0.059 g, 0.25 mmol). Then, the mixture was stirred at room temperature for 10s. The obtained powder was filtered, washed with methanol, and exchanged with methanol for two days.

Powder synthetic method 2: A preheated ethanol solution (8.0 mL) of 4,4′-azopyridine (0.095 g, 0.5 mmol) was dropped into a preheated ethylene glycol solution (8.0 mL) of $(NH_4)_2GeF_6$ (0.056 g, 0.25 mmol) and $Cu(BF_4)_2 \cdot xH_2O$ (0.059 g, 0.25 mmol). Then, the mixture was heated at 65 ºC for 24 h under stirring. The obtained powder was filtered, washed with methanol, and exchanged with methanol for two days.

Preparation of SIFSIX-1-Cu (Cu(4, 4'-bipyridine)$_2$SiF$_6$·8H$_2$O)n

4,4′-bipyridine (0.35 g, 2.24 mmol) was dissolved in 40 mL ethylene glycol at 343 K and an aqueous solution 20 mL of $Cu(BF_4)_2 \cdot xH_2O$ (0.266 g, 1.12 mmol) and $(NH_4)_2SiF_6$ (0.199 g, 1.12 mmol) was added to the former solution. The mixture was then heated at 70 °C for 3 h with stirring. The obtained purple powder was washed with methanol, and exchanged with methanol for 1 day[2,3].

Preparation of SIFSIX-2-Cu-i (Cu(4,4′-bipyridylacetylene)$_2$SiF$_6$)

A methanol solution (4.0 mL) of 4,4′-bipyridylacetylene (0.052 g, 0.286 mmol) was mixed with an aqueous solution (4.0 mL) of $(NH_4)_2SiF_6$ (0.046 g, 0.26 mmol) and $Cu(BF_4)_2 \cdot xH_2O$ (0.062 g, 0.26 mmol). Then, the mixture was heated at 80 °C for 12 h. The obtained powder was filtered, washed with methanol, and exchanged with methanol for 3 days[2,3].

Preparation of TIFSIX-2-Cu-i (Cu(4,4′-bipyridylacetylene)$_2$TiF$_6$)

A methanol solution (4.0 mL) of 4,4′-bipyridylacetylene (0.052 g, 0.286 mmol) was mixed with an aqueous solution (4.0 mL) of $(NH_4)_2TiF_6$ (0.052 g, 0.26 mmol) and $Cu(BF_4)_2 \cdot xH_2O$ (0.062 g, 0.26 mmol). Then, the mixture was heated at 80 °C for 12 h. The obtained powder was filtered, washed with methanol, and exchanged with methanol for 3 days[2,3].

Preparation of SIFSIX-3-Ni (Ni(pyrazine)$_2$SiF$_6$)n

A methanol solution (20 mL) of $(NH_4)_2SiF_6$ (0.178 g, 1 mmol), $Ni(BF_4)_2$ (0.232 g, 1 mmol) and pyrazine (0.161 g, 2 mmol) was mixed and heated at 85 °C for 3 days. The obtained sky blue powder was washed with methanol. Then exchanged with methanol for 1 days[2,3].

Single crystal X-ray diffraction

Single crystal X-ray diffraction data for ZU-33 were collected on a Bruker D8 VENTURE diffractometer equipped with a PHOTONII/CMOS detector (GaK_{α}, λ =1.314139 Å). Indexing was performed using APEX3. Data integration and reduction were completed using SaintPlus 6.01. Absorption correction was performed by the multi-scan method implemented in SADABS. The space group was determined using XPREP implemented in APEX3. The structure was solved with SHELXS-2018 (direct methods) and refined on F2(nonlinear least-squares method) with SHELXL-2018 contained in APEX3 program packages. All non-hydrogen atoms were refined anisotropically. Firstly, the ZU-33 single crystals were loaded into the sample tube for adsorption test, the inner wall of the sample tube was coated with oil (polybutenes), which slowly flowed down the tube wall. Then, C_2H_2 and C_3H_4 gas was backfilled, respectively. After ZU-33 single crystals were sealed by oil, they were taken out and tested on a Bruker D8 VENTURE diffractometer.

Powder X-ray diffraction

Powder x-ray diffraction (PXRD) data were collected using a SHIMADZU XRD-600 diffractometer (Cu K_{α}, λ = 1.540598 Å) with an operating power of 40 KV, 40 mA and a scan speed of 4.0 ° min^{-1}. The data were collected in the range of 2θ = 5° to 40°.

Gas adsorption measurements

ZU-33 was evacuated at 65 ºC for 12 h until the pressure dropped below 7 µm Hg. The single-component adsorption isotherms of CH_4, C_2H_2, C_2H_4, C_2H_6, C_3H_4, C_3H_4 (PD) and C_3H_6 were collected at 298~308 K on activated ZU-33 using ASAP 2460 Analyzer (Micromeritics). The single-component adsorption isotherms of C_4H_6, *n*-C_4H_8 and *i*-C_4H_8 were collected at 298~308 K on activated ZU-33 using ASAP 2050 Analyzer (Micromeritics).

Breakthrough experiments

The breakthrough experiments were carried out in a dynamic gas breakthrough equipment[2]. All experiments were conducted using a stainless steel column (4.6 mm inner diameter × 50 mm). According

to the different particle size and density of the sample powder, the weight packed in the column was: 0.332 g for ZU-33, 0.3 g for SIFSIX-1-Cu, 0.21 g for SIFSIX-2-Cu-i, 0.242 g for TIFSIX-2-Cu-i and 0.26 g for SIFSIX-3-Ni. The mixed gas of $H_2/CH_4/C_2H_2/C_2H_4/C_2H_6/C_3H_4/C_3H_4$(PD)/$C_3H_6/C_4H_6$/*n*-$C_4H_8$/*i*-$C_4H_8$ = 15.57/30/1.02/34.3/6.99/0.3/0.28/8.93/1.48/0.42/0.71 (v/v/v/v/v/v/v/v/v/v/v) was introduced at a total flow rate of 2.0 mL min^{-1} at 298 K and 303 K. Outlet gas from the column was monitored using gas chromatography (GC-490) with the thermal conductivity detector TCD. After breakthrough experiment, the adsorption bed was regenerated by N_2 flow (10 mL min^{-1}) for 1 hour at 323 K or by vacuuming for about 30 min at 323 K.

Computational Methods

Simulation details. All calculations were performed using the combination of first-principle density functional theory (DFT) and plane-wave ultrasoft pseudopotentil implemented in the Materials Studio, CASTEP code[4]. A semi-empirical addition of dispersive forces to conventional DFT was included in the calculation to account for van der Waals interactions. Calculations were performed under the generalized gradient approximation (GGA) with Perdew-Burke-Ernzerhof (PBE) exchange correlation. A cutoff energy of 544 eV and a 2×2×4 k-point mesh with smearing 0.2 ev were found to be enough for the total energy to converge within 1×10^{-6} ev/atom. Notably, in all the simulation process, the structures were optimized with a full structural relaxation that allowed all atomic positions and unit cell parameters to vary.

The energy barrier. The periodic slab models with periodic boundary conditions were sued to represent ZU-33 surface. The vacuum region between slabs was 20 Å to eliminate spurious interactions between the guest molecules with the periodic image of the bottom layer of the surface. The surface model and the host structure would be would be first optimized using the experimentally-obtained single crystal structures as initial geometries with a full structural relaxation. The isolated guest molecules (C_2H_2, C_2H_4, C_3H_4 and C_3H_4 (PD)) were placed in a supercell (with the same cell dimensions as the MOF crystal) and also relaxed as references. Then, the guest molecules were introduced onto the host surface and different

locations in the channel pore of the host structure, respectively, followed by a full structural relaxation. And the optimized configurations having the lowest energy were used for the subsequent analysis and calculation. The transition state search calculations were used to capture the transition statues associated with guest transport between the two know energy minimum configurations from the host surface to the channel pore. The initial state I was defined as the optimized guest-free host and optimized guest, and the system energy was set as the reference. The state II was defined as the optimized host-guest structures where guests were introduced onto the host surface, the state III was defined as the transition state and the state IV was defined as the optimized host-guest structures where guests were introduced into the channel pore. The energy barrier was calculated using:

$$\Delta E' = \text{E (state III)} - \text{E (state II)} \qquad (1)$$

where E (state III) is the energy of the transition state, E (state II) is the energy of the optimized host-guest structures where guests were introduced onto the host surface.

The static binding energy. The static binding energy (at T=0 K) was calculated using:

$$\Delta E = \text{E (MOF)} + \text{E (gas)} - \text{E (MOF+ gas)} \qquad (2)$$

where E (MOF) is the energy of the optimized guest-free host, E (gas) is the energy of the optimized guest and E (MOF+ gas) is the total energy of the optimized host-guest structures.

Supplementary Figures

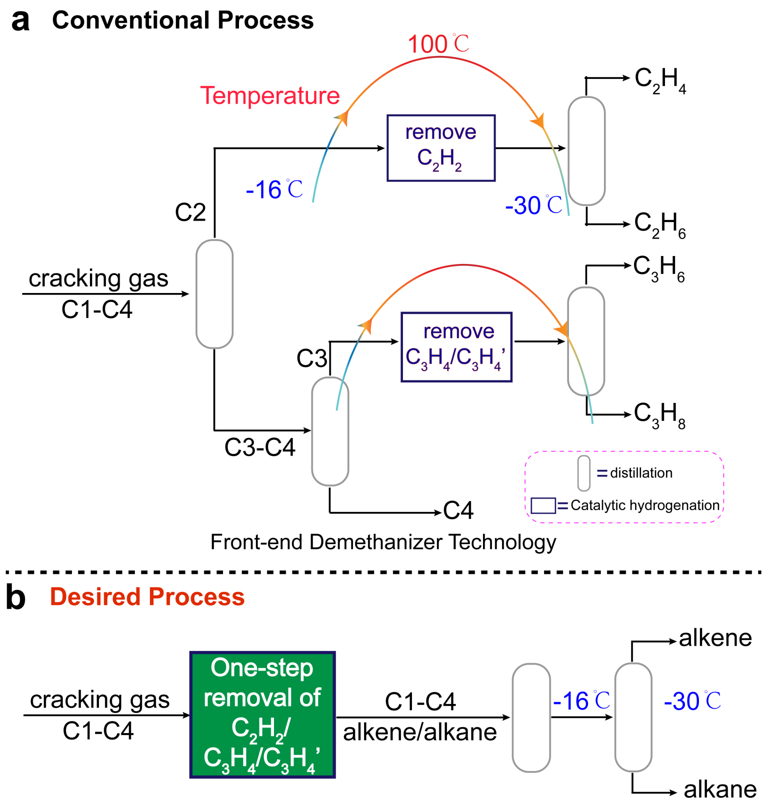

Supplementary Figure 1. The scheme of alkynes and propadiene removal process. (a) The conventional removal process (Front-end Demetanizer Process), in which both C2 and C3 parts need to undergo the combined process of hydrogenation and distillation to realize the depth-removal of alkynes and propadiene. Due to the distinct operation conditions of the two continuous process, the separation involves the "parabola" like sudden temperature change, like C2 part, the temperature in hydrogenation process is ca 100 °C, while in distillation column, it is -16/-30 °C, thus, a substantial energy input is required to realize this process. **(b)** The desired removal process, in which one-step removal of alkynes and propadiene before the cracking gas mixture enters into the distillation tower.

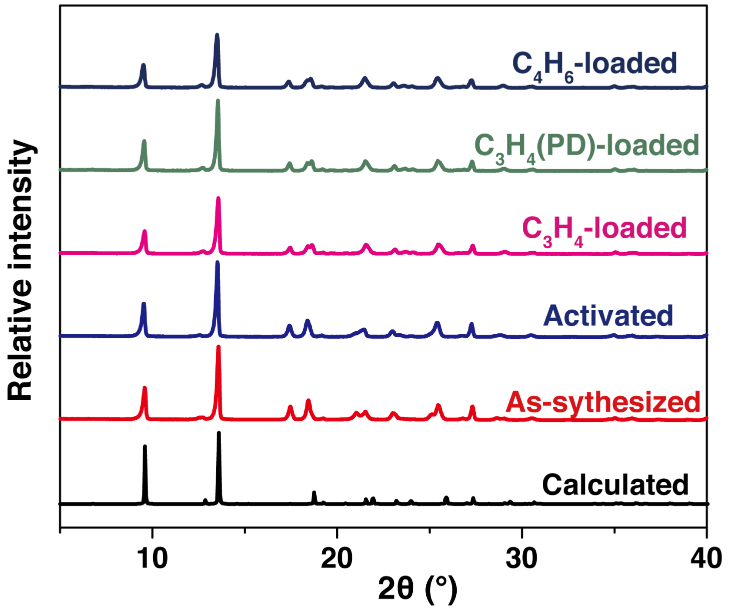

Supplementary Figure 2. The powder X-ray diffraction patterns of ZU-33.

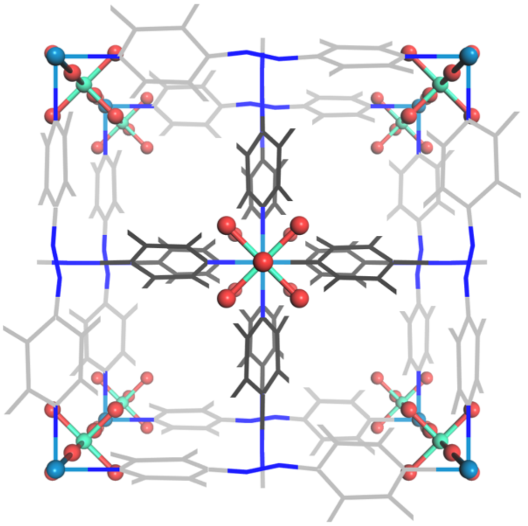

Supplementary Figure 3. The crystal structure of ZU-33. Color code: F, red; Ge, light green; Cu, light blue; C, gray- 40%; H, gray-25%; N, blue. The interpenetrated nets are highlighted in dark.

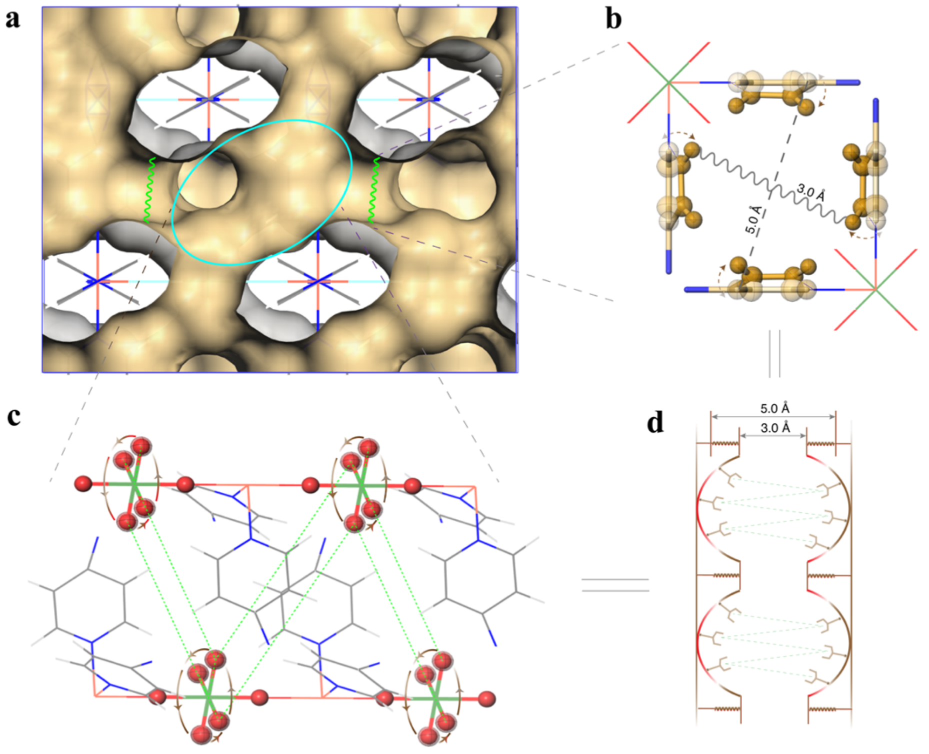

Supplementary Figure 4. The connolly surface of ZU-33 channel (**a**). The responsive bottleneck of ZU-33 and the rotation of the pyridrine rings (**b**), the '*ziazag'* shaped high-density anions in the channel and the rotation of the F anions (**c**). Schematic illustration the 'gourd type' channel structure of ZU-33 (**d**).

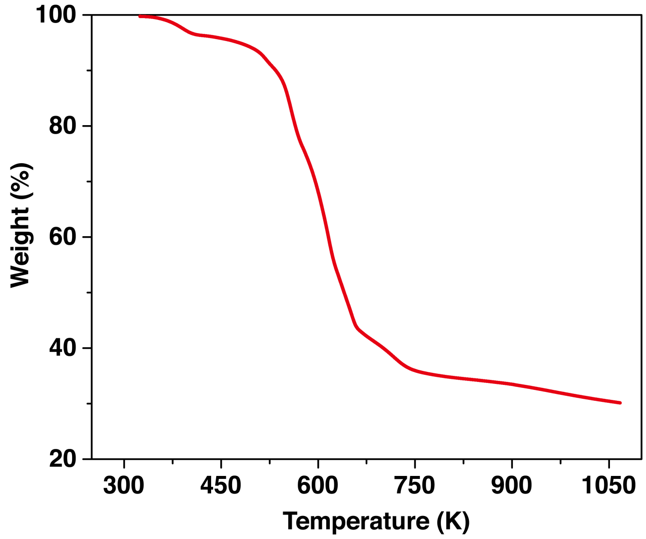

Supplementary Figure 5. The TGA curve of ZU-33.

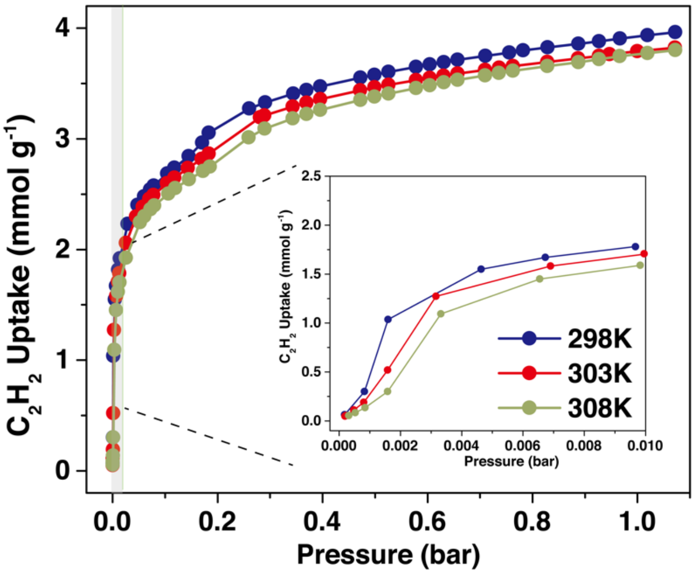

Supplementary Figure 6. The adsorption isotherms of C_2H_2 on ZU-33 at 298, 303 and 308 K in two pressure regions, 0-1.0 bar and 0-0.01 bar (the inset).

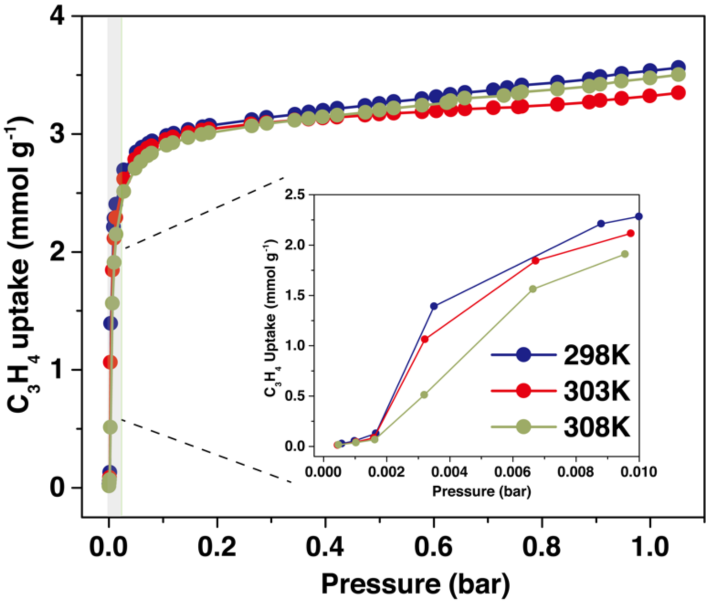

Supplementary Figure 7. The adsorption isotherms of C_3H_4 on ZU-33 at 298, 303 and 308 K in two pressure regions, 0-1.0 bar and 0-0.01 bar (the inset).

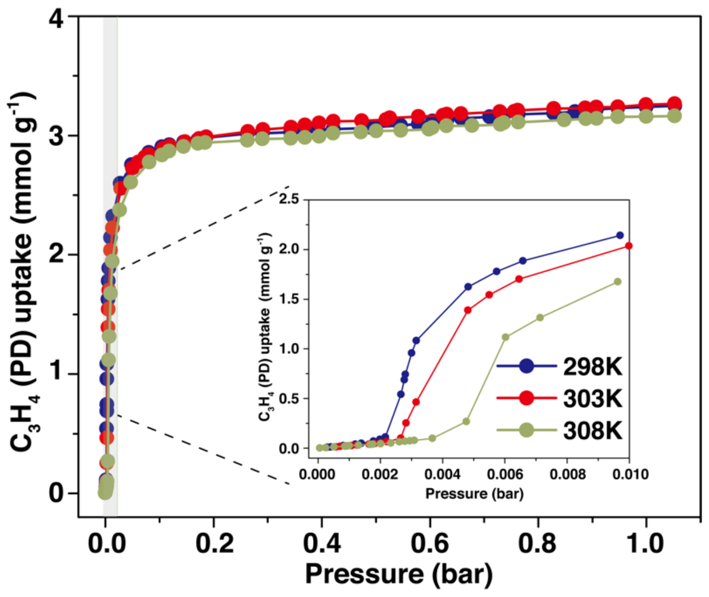

Supplementary Figure 8. The adsorption isotherms of C_3H_4 (PD) on ZU-33 at 298, 303 and 308 K in two pressure regions, 0-1.0 bar and 0-0.01 bar (the inset).

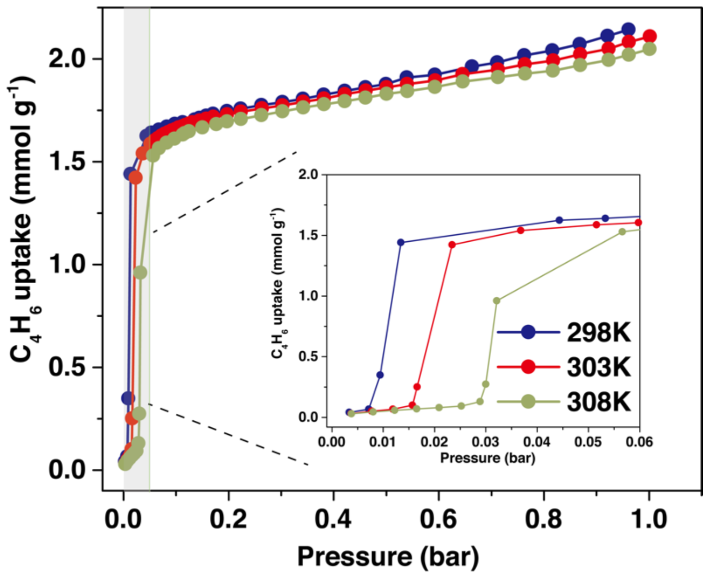

Supplementary Figure 9. The adsorption isotherms of C_4H_6 on ZU-33 at 298, 303 and 308 K in two pressure regions, 0-1.0 bar and 0-0.01 bar (the inset).

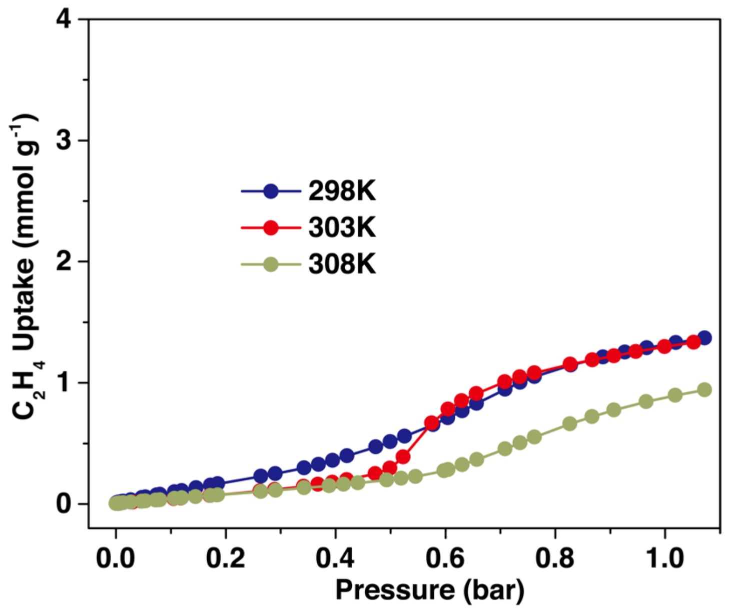

Supplementary Figure 10. The adsorption isotherms of C_2H_4 on ZU-33 at 298, 303 and 308 K in 0-1.0 bar.

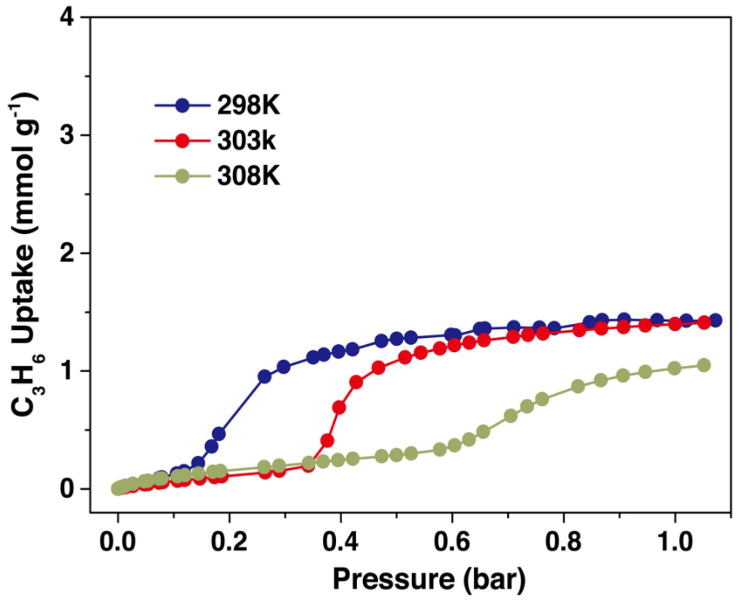

Supplementary Figure 11. The adsorption isotherms of C_3H_6 on ZU-33 at 298, 303 and 308 K in 0-1.0 bar.

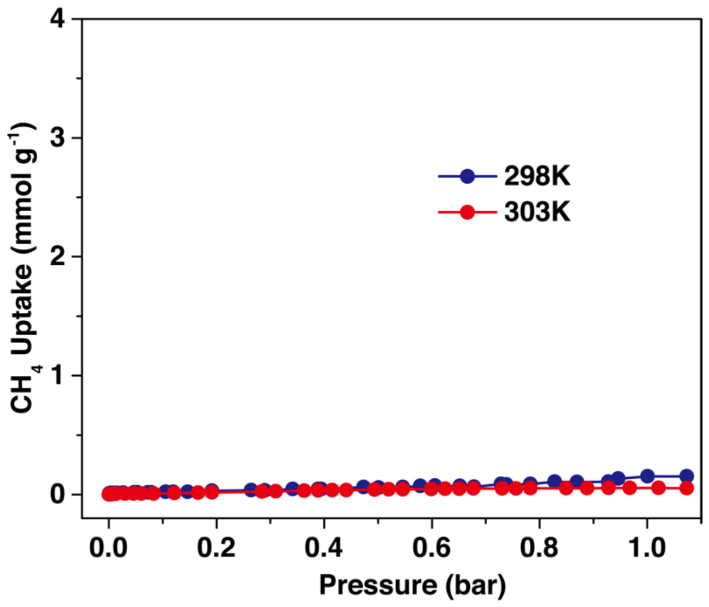

Supplementary Figure 12. The adsorption isotherms of CH_4 on ZU-33 at 298 and 303 K in 0-1.0 bar.

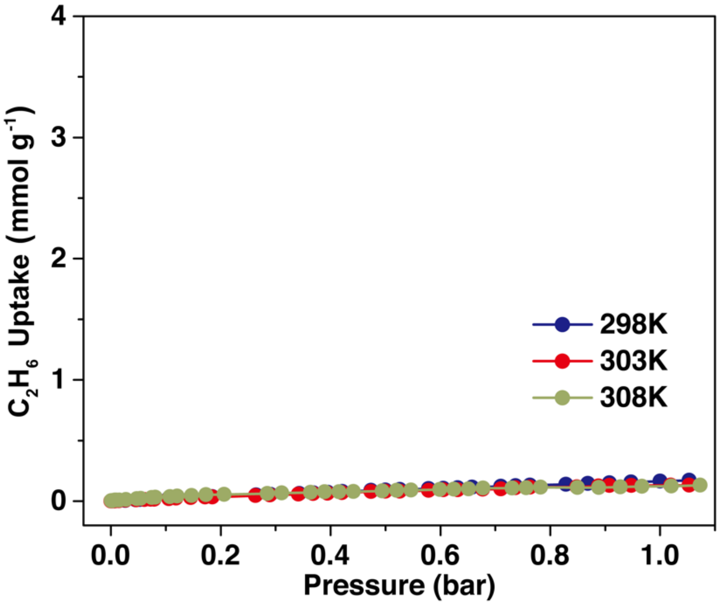

Supplementary Figure 13. The adsorption isotherms of C_2H_6 on ZU-33 at 298, 303 and 308 K in 0-1.0 bar.

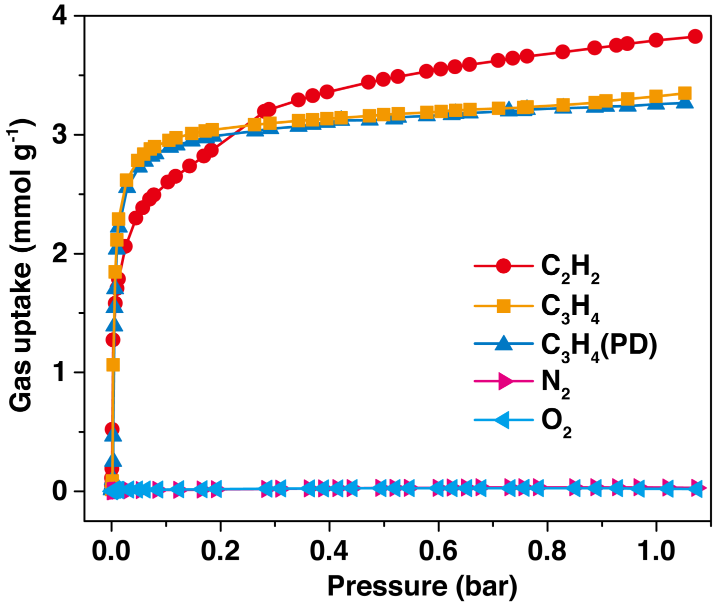

Supplementary Figure 14. The adsorption isotherms of C_2H_2, C_3H_4, C_3H_4 (PD), N_2 and O_2 on ZU-33 at 303 K in 0-1.0 bar.

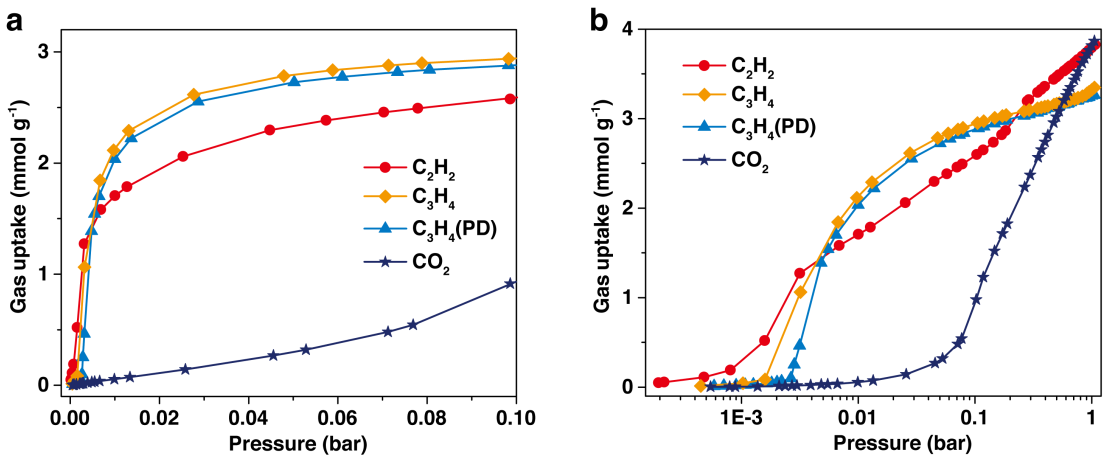

Supplementary Figure 15. The adsorption isotherms of C_2H_2, C_3H_4, C_3H_4 (PD) and CO_2 on ZU-33 at 303 K in 0-0.1 bar (a), 0-1.0 bar (b).

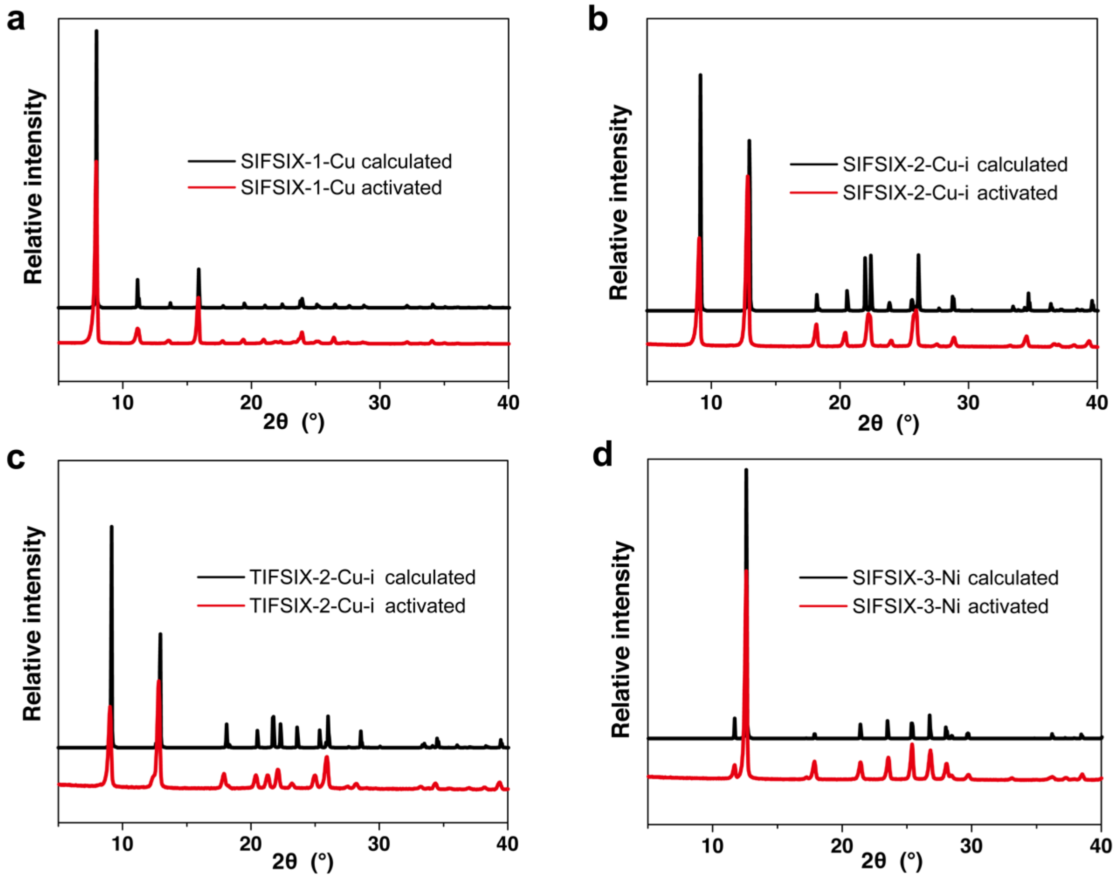

Supplementary Figure 16. The powder X-ray diffraction patterns of SIFSIX-1-Cu (**a**), SIFSIX-2-Cu-i (**b**), TIFSIX-2-Cu-i (**c**) and SIFSIX-3-Ni (**d**).

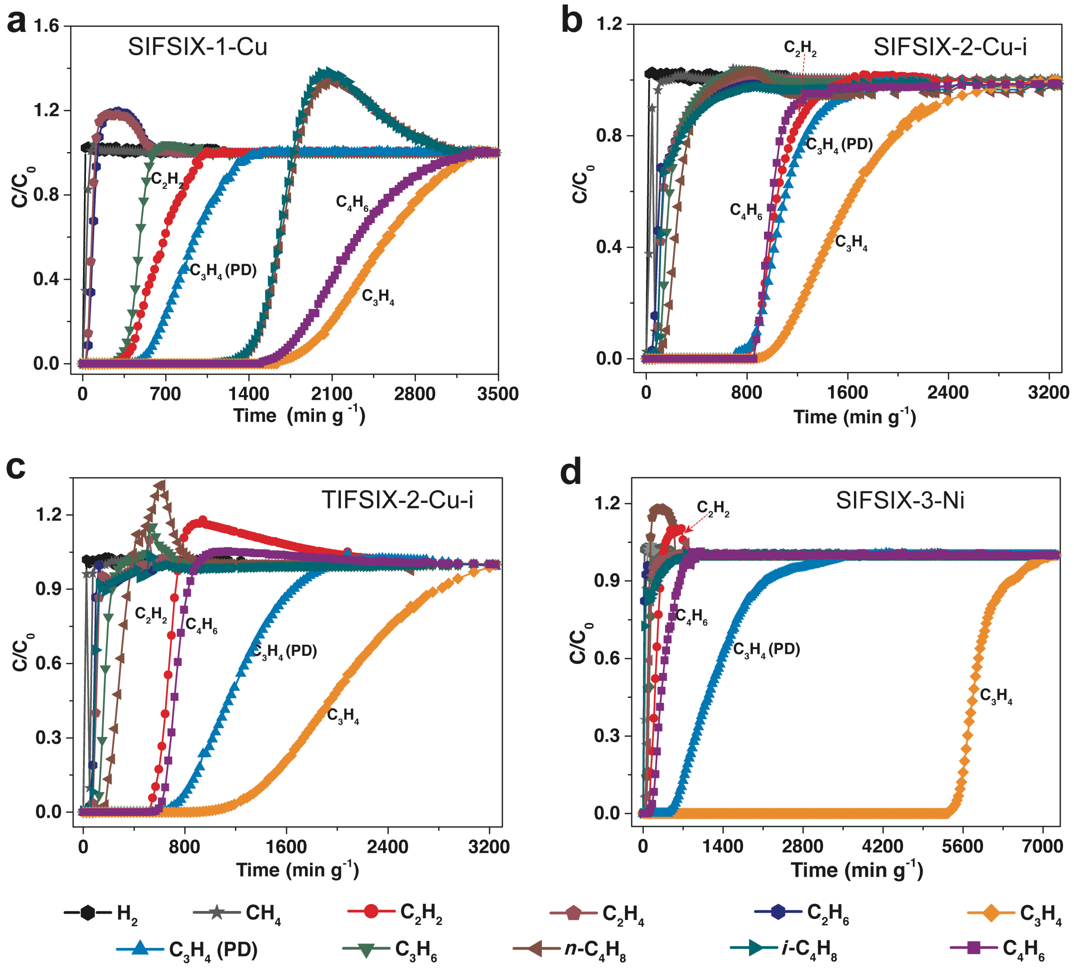

Supplementary Figure 17. Experimental column breakthrough curves for the mixture of H_2/CH_4/C_2H_2/C_2H_4/C_2H_6/C_3H_4/C_3H_4 (PD)/C_3H_6/C_4H_6/*n*-C_4H_8/*i*-C_4H_8 on SIFSIX-1-Cu (**a**), SIFSIX-2-Cu-i (**b**), TIFSIX-2-Cu-i (**c**) and SIFSIX-3-Ni (**d**) at 298 K.

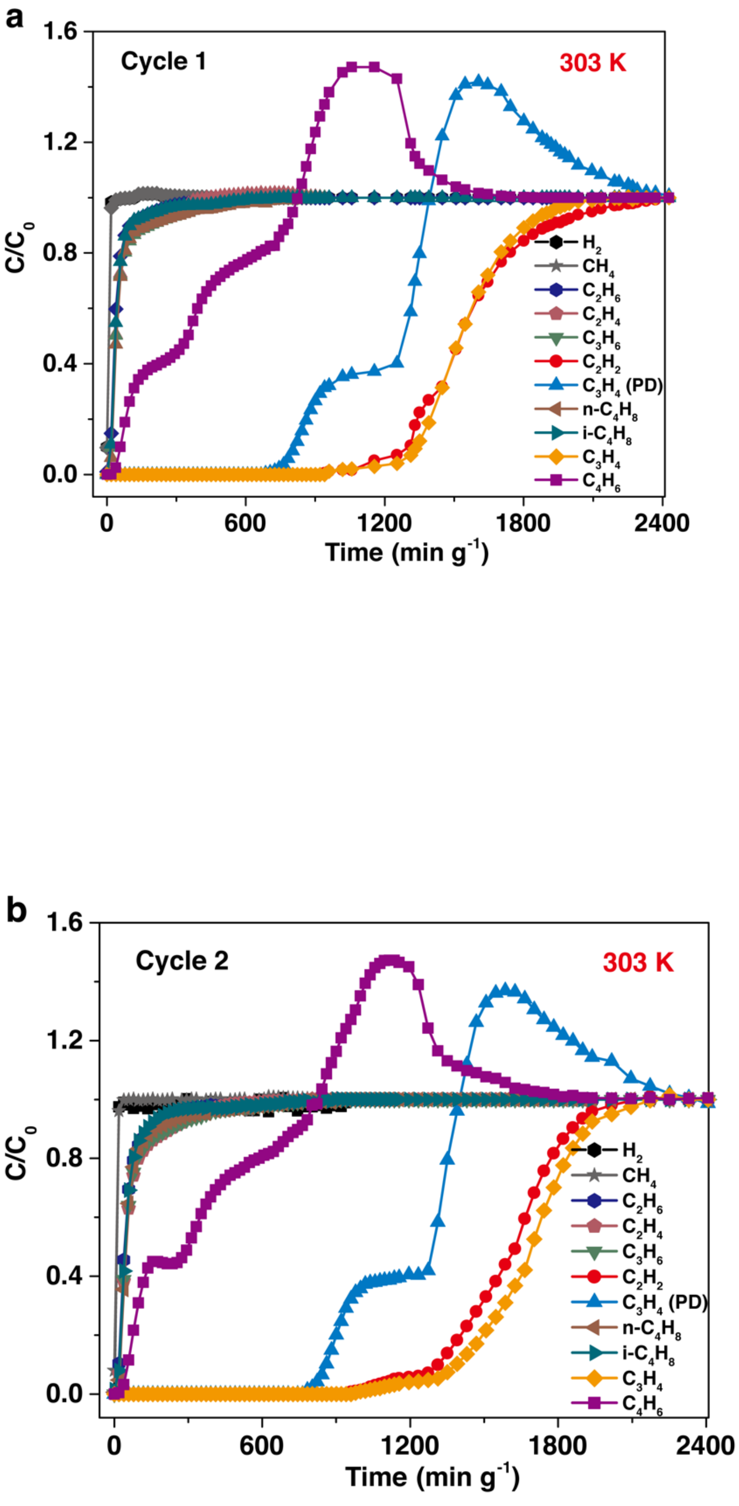
a
Cycle 1
303 K
C/C0
Time (min g-1)
H2
CH4
C2H6
C2H4
C3H6
C2H2
C3H4 (PD)
n-C4H8
i-C4H8
C3H4
C4H6
b
Cycle 2
303 K
C/C0
Time (min g-1)
H2
CH4
C2H6
C2H4
C3H6
C2H2
C3H4 (PD)
n-C4H8
i-C4H8
C3H4
C4H6

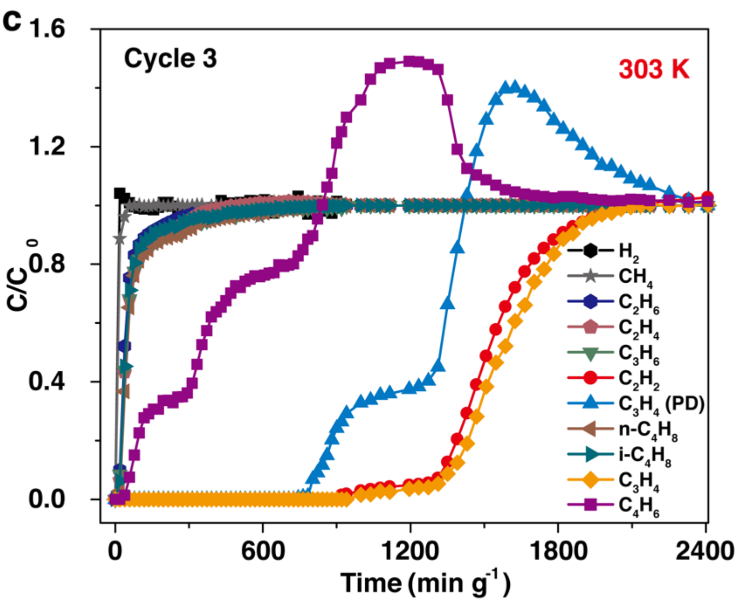
c
Cycle 3
303 K
C/C0
Time (min g-1)
H2
CH4
C2H6
C2H4
C3H6
C2H2
C3H4 (PD)
n-C4H8
i-C4H8
C3H4
C4H6
0.0
0.4
0.8
1.2
1.6
0
600
1200
1800
2400

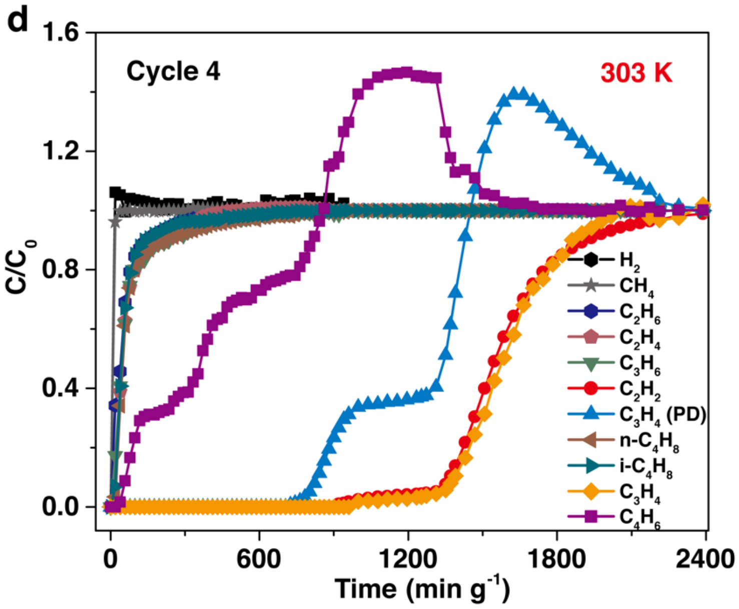
d
Cycle 4
303 K
C/C0
Time (min g-1)
H2
CH4
C2H6
C2H4
C3H6
C2H2
C3H4 (PD)
n-C4H8
i-C4H8
C3H4
C4H6
0.0
0.4
0.8
1.2
1.6
0
600
1200
1800
2400

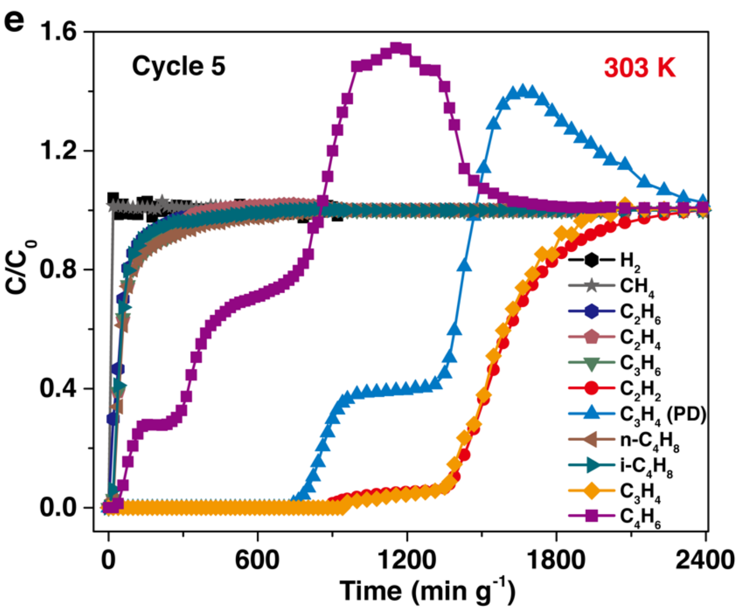

Supplementary Figure 18. (**a-e**) Cycling breakthrough curves for the mixture of $H_2/CH_4/C_2H_2/C_2H_4/C_2H_6/C_3H_4/C_3H_4$ (PD)/C_3H_6/C_4H_6/*n*-C_4H_8/*i*-C_4H_8 on ZU-33 at 303 K over five cycles.

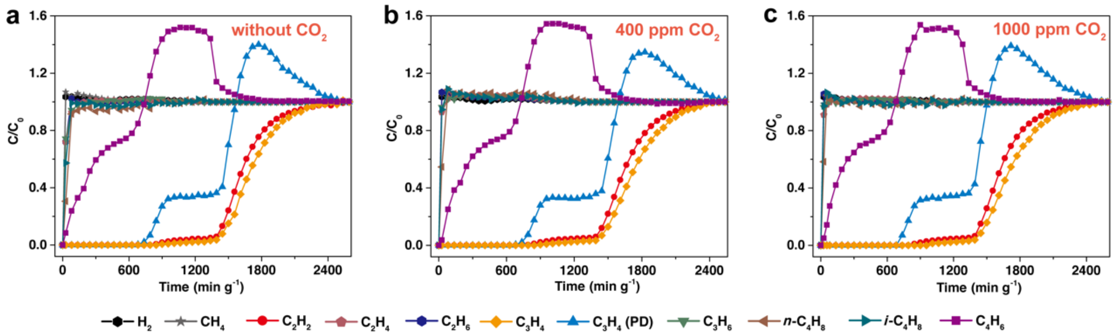

Supplementary Figure 19. Experimental column breakthrough curves for the mixture of $H_2/CH_4/C_2H_2/C_2H_4/C_2H_6/C_3H_4/C_3H_4$ (PD)/C_3H_6/C_4H_6/*n*-C_4H_8/*i*-C_4H_8 on ZU-33 with different contents of CO_2 at 303 K.

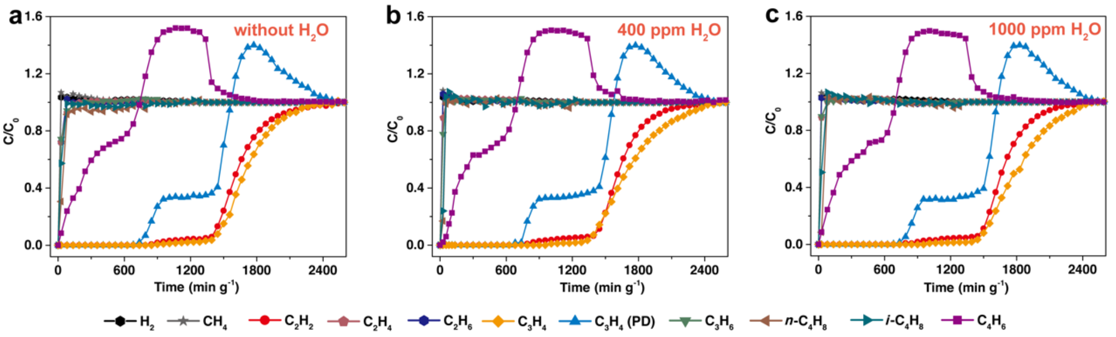

Supplementary Figure 20. Experimental column breakthrough curves for the mixture of $H_2/CH_4/C_2H_2/C_2H_4/C_2H_6/C_3H_4/C_3H_4$ (PD)/C_3H_6/C_4H_6/n-C_4H_8/i-C_4H_8 on ZU-33 with different contents of H_2O at 303 K.

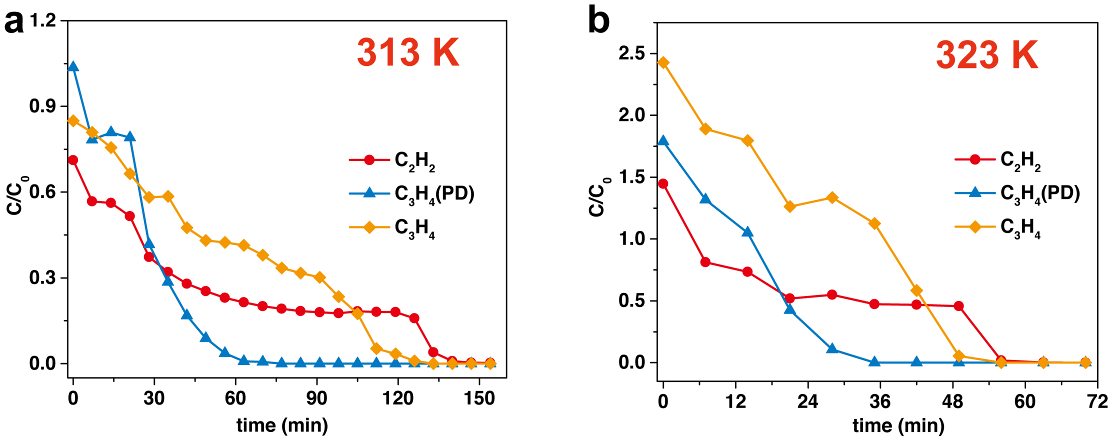

Supplementary Figure 21. Experimental desorption curves for C_2H_2, C_3H_4 (PD), and C_3H_4 on ZU-33 under different temperatures. Before desorption, the breakthrough experiment for the mixture of $H_2/CH_4/C_2H_2/C_2H_4/C_2H_6/C_3H_4/C_3H_4$ (PD)/C_3H_6/C_4H_6/n-C_4H_8/i-C_4H_8 was carried out on ZU-33 at 303 K.

a

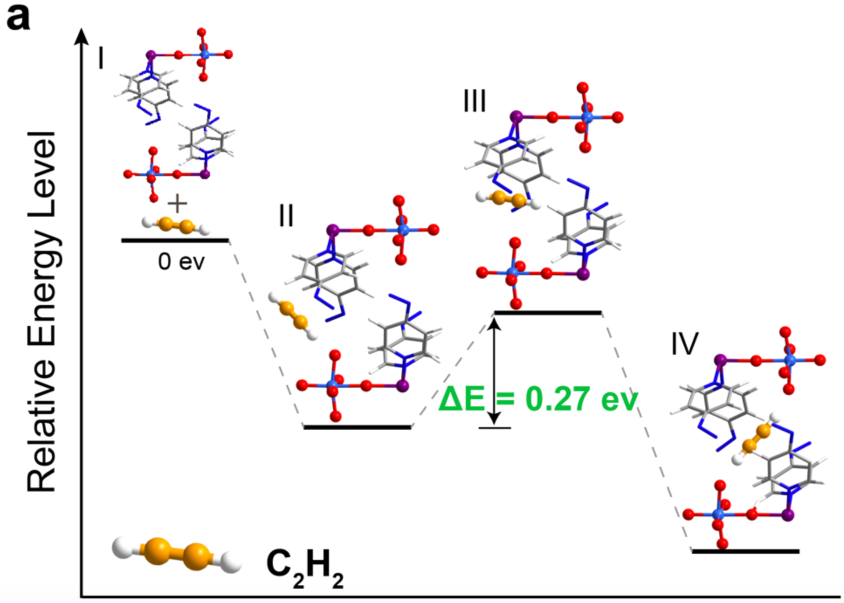
Relative Energy Level
I
+
0 ev
II
III
ΔE = 0.27 ev
IV
C2H2

b

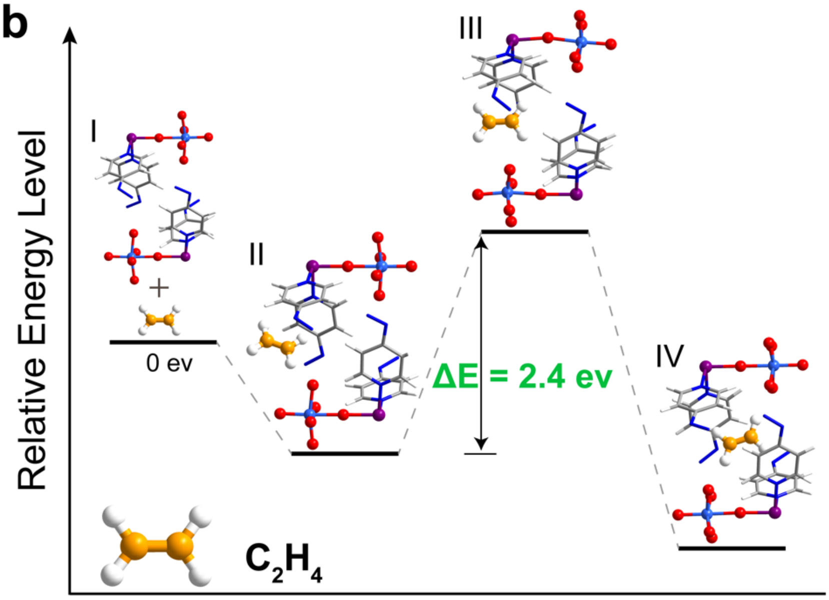
Relative Energy Level
I
+
0 ev
II
III
ΔE = 2.4 ev
IV
C2H4

c

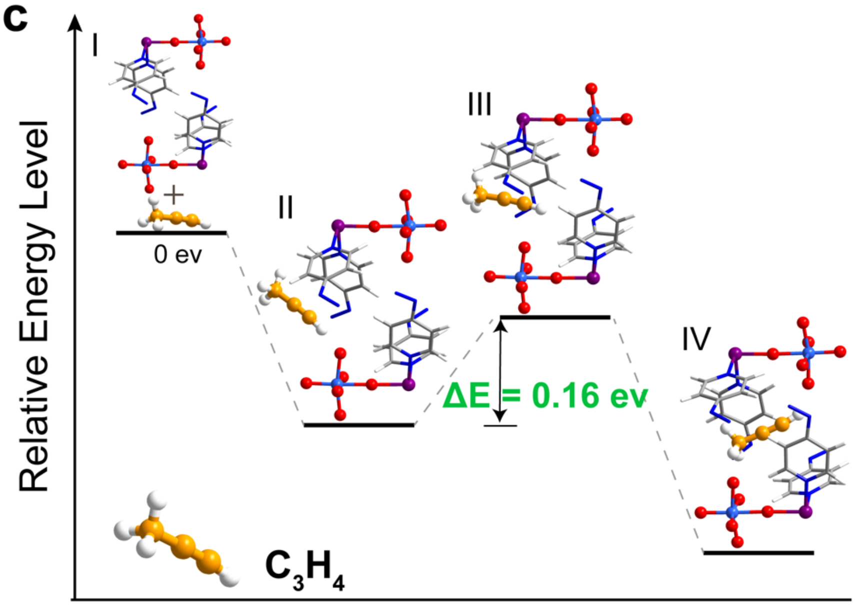
Relative Energy Level
I
+
0 ev
II
III
ΔE = 0.16 ev
IV
C_3H_4

d

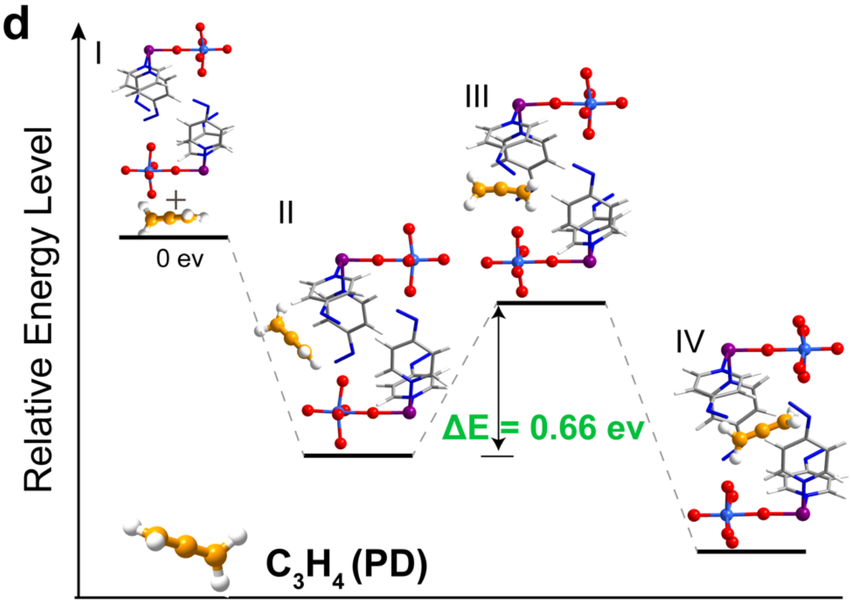
Relative Energy Level
I
+
0 ev
II
III
ΔE = 0.66 ev
IV
C_3H_4 (PD)

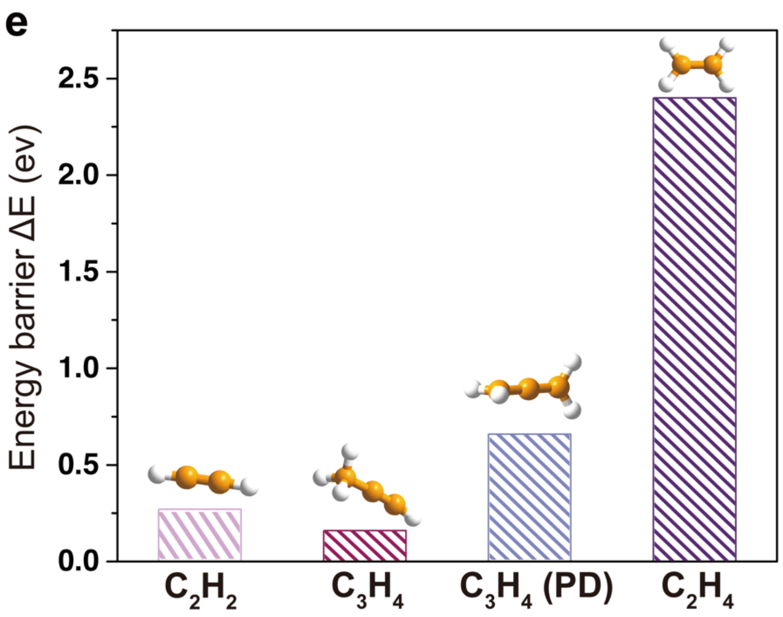

Supplementary Figure 22. Illustration of the interaction energy pathway and the corresponding energy levels for C_2H_2 (**a**), C_2H_4 (**b**), C_3H_4 (**c**) and C_3H_4 (PD) (**d**). Comparison of energy barriers of C_2H_2, C_3H_4, C_2H_4 and C_3H_4 (PD) (**e**). Color code: F, red; Ge, light blue; Cu, modena; C (in framework), gray; C (in hydrocarbons), orange; H, withe; N, blue.

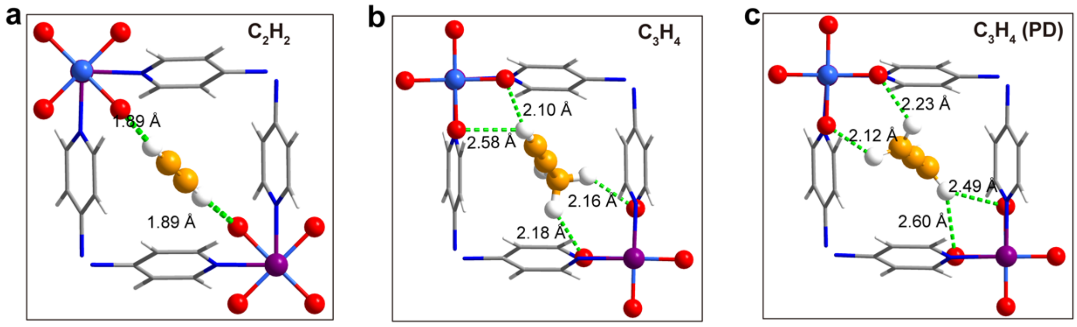

Supplementary Figure 23. DFT-D calculated host-guest interactions, C_2H_2 (**a**), C_3H_4 (**b**) and C_3H_4 (PD) (**c**). Color code: F, red; Ge, light blue; Cu, modena; C (in framework), gray; C (in hydrocarbons), orange; H, withe; N, blue.

Supplementary Tables

Supplementary Table 1. Crystal Structure data and refinement conditions for degassed ZU-33.

Unit cell parameters	
Formula sum	C20 H16 Cu F6 Ge N8
Formula weight	618.54 g/mol
Crystal system	tetragonal
Space –group	P 4/m m m (123)
Cell parameters	a=13.033(2) Å c=8.102(3) Å
Cell ratio	a/b=1.0000 b/c=1.6086 c/a=0.6217
Cell volume	1376.2(7) $Å^3$
Z	2
Calc.density	1.49259 g/cm^3

Supplementary Table 2. Crystal Structure data and refinement conditions for C_2H_2-loaded ZU-33.

Unit cell parameters	
Formula sum	C21 H17 Cu F6 Ge N8
Formula weight	631.56 g/mol
Crystal system	tetragonal
Space –group	P 4/m n c (128)
Cell parameters	a=13.071(3) Å c=8.406(2) Å
Cell ratio	a/b=1.0000 b/c=1.5550 c/a=0.6431
Cell volume	1436.17(70) $Å^3$
Z	2
Calc.density	1.46036 g/cm^3

Supplementary Table 3. Crystal Structure data and refinement conditions for C_3H_4-loaded ZU-33.

Unit cell parameters	
Formula sum	C26 H24 Cu F6 Ge N8
Formula weight	698.66 g/mol
Crystal system	tetragonal
Space –group	P 4/n m m (129)
Cell parameters	a=13.046(3) Å c=8.3457(18) Å
Cell ratio	a/b=1.0000 b/c=1.5632 c/a=0.6397
Cell volume	1420.42(60) $Å^3$
Z	2
Calc.density	1.63343 g/cm^3

Supplementary References

1. Zhang, Z. et al. Sorting of C4 olefins with interpenetrated hybrid ultramicroporous materials by combining molecular recognition and size-sieving. *Angew. Chem. Int. Ed*. **56**, 16282–16287 (2017).

2. Cui, X. L. et al. Pore chemistry and size control in hybrid porous materials for acetylene capture from ethylene. *Science* **353**, 141-144 (2016).

3. Nugent, P. et al. Porous materials with optimal adsorption thermodynamics and kinetics for CO_2 separation. *Nature* **495**, 80-84 (2013).

4. Segall, M. D. et al. First-principles simulation: ideas, illustrations and the CASTEP code. *J. Physics-Condens. Matter.* **14**, 2717–2744 (2002).
